# Chiral antiferromagnetic Josephson junctions as spin-triplet supercurrent spin valves and d.c. SQUIDs

**DOI:** 10.1038/s41565-023-01336-z

**Published:** 2023-03-30

**Authors:** Kun-Rok Jeon, Binoy Krishna Hazra, Jae-Keun Kim, Jae-Chun Jeon, Hyeon Han, Holger L. Meyerheim, Takis Kontos, Audrey Cottet, Stuart S. P. Parkin

**Affiliations:** 1 Max Planck Institute of Microstructure Physics, Halle (Saale), Germany; 2 Department of Physics, Chung-Ang University (CAU), Seoul, Republic of Korea; 3 Laboratoire de Physique de l’Ecole normale supérieure, ENS, Université PSL, CNRS, Sorbonne Université, Université de Paris, Paris, France

**Keywords:** Superconducting properties and materials, Spintronics

## Abstract

Spin-triplet supercurrent spin valves are of practical importance for the realization of superconducting spintronic logic circuits. In ferromagnetic Josephson junctions, the magnetic-field-controlled non-collinearity between the spin-mixer and spin-rotator magnetizations switches the spin-polarized triplet supercurrents on and off. Here we report an antiferromagnetic equivalent of such spin-triplet supercurrent spin valves in chiral antiferromagnetic Josephson junctions as well as a direct-current superconducting quantum interference device. We employ the topological chiral antiferromagnet Mn_3_Ge, in which the Berry curvature of the band structure produces fictitious magnetic fields, and the non-collinear atomic-scale spin arrangement accommodates triplet Cooper pairing over long distances (>150 nm). We theoretically verify the observed supercurrent spin-valve behaviours under a small magnetic field of <2 mT for current-biased junctions and the direct-current superconducting quantum interference device functionality. Our calculations reproduce the observed hysteretic field interference of the Josephson critical current and link these to the magnetic-field-modulated antiferromagnetic texture that alters the Berry curvature. Our work employs band topology to control the pairing amplitude of spin-triplet Cooper pairs in a single chiral antiferromagnet.

## Abstract (web summary)

A Josephson junction with a weak link made from the chiral antiferromagnet Mn_3_Ge can act as a spin-triplet supercurrent spin valve.

## Main

Intensive studies in coupling superconducting condensate state with magnetic-exchange spin splitting have opened up a research field of superconducting spintronics^1–3^, which promises to realize dissipationless spin-based logic and memory technologies. Of particular relevance in this research is the theoretical prediction^4–7^ and experimental verification^8–12^ that spin-polarized triplet pairing states can be created via spin-mixing and spin-rotation processes (for example, non-collinear exchange fields^4–6,8–10^ in real space and/or spin–orbit fields^7,11,12^ in reciprocal/*k* space) at proximity-engineered superconductor/ferromagnet (FM) interfaces^1–12^. With these advances, the field of superconducting spintronics involving spin-polarized triplet Cooper pairs^1–3^ can answer two practical aspects: how to efficiently generate such triplet pairs and how to tune them in a controllable manner. Yet, as previously pointed out^4,13,14^, fulfilling these two requirements at the same time seems challenging because the preconfigured robust non-collinearity of spin-mixer and spin-rotator magnetizations for a higher singlet-to-triplet pair conversion makes it difficult to control by an external stimulus.

The active and reversible control of spin-polarized triplet supercurrents has so far been mostly achieved in ferromagnetic Josephson junctions (JJs)^13,14^, where at least three FMs constitute a Josephson barrier whose relative magnetization directions, and therefore the non-collinearity, can be controlled by external magnetic fields^4,13,14^. This has led to the so-called spin-triplet supercurrent spin valve^4,13,14^, in which the proximity-created spin-polarized triplet supercurrents can be switched on and off. However, the fabrication of such ferromagnetic spin-triplet JJs^13,14^ requires delicate interface engineering, for instance, electronic energy band matching between neighbouring layers, selectivity in coercive fields of the spin-mixer and spin-rotator FMs and circumventing the out-of-plane (OOP) component of stray magnetic fields.

**Fig. 1:**
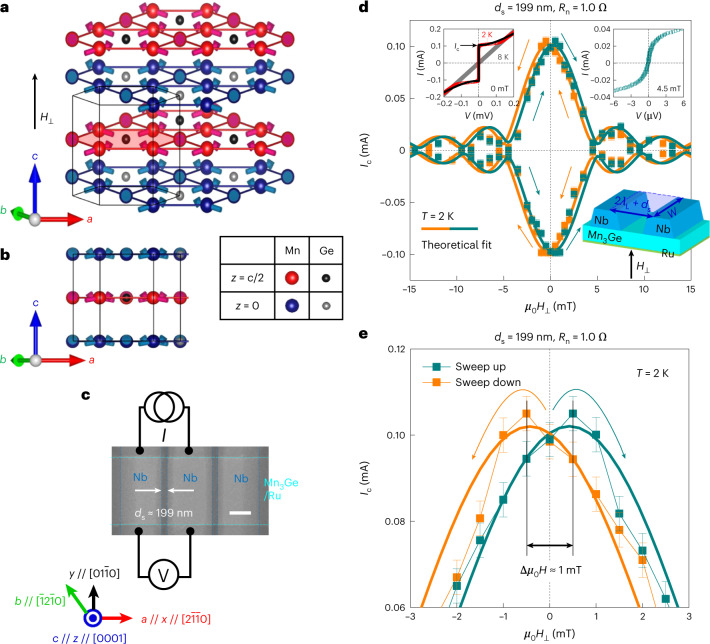
Hysteretic magnetic-field interference pattern of chiral antiferromagnetic spin-triplet JJs. **a**,**b**, Probable 120° chiral antiferromagnetic configurations and crystal structure of *D*0_19_-Mn_3_Ge(0001) canted out of the kagome plane under a perpendicular magnetic field *μ*_0_*H*_⊥_ (perpendicular to the *a*–*b* plane). Two layers of Mn and Ge atoms are stacked along the *c* axis (parallel to the *z* axis), where blue and grey (red and black circles) represent Mn and Ge atoms lying in the *z* = 0 (*z* = *c*/2) plane, respectively. **c**, Scanning electron micrograph of the fabricated Nb/Mn_3_Ge/Nb lateral JJs. Note that the 5-nm-ultrathin Ru underlayer acts as a buffer layer (Methods). Scale bar, 0.5 µm. **d**, Josephson critical current *I*_c_ versus *μ*_0_*H*_⊥_ plot for the *d*_s_ = 199 nm Nb/Mn_3_Ge/Nb JJ, taken at *T* = 2 K. Here *μ*_0_*H*_⊥_ is applied perpendicular to the kagome (interface) plane of the Mn_3_Ge barrier (Nb electrodes) (bottom inset). The top-left inset displays the zero-field *I*–*V* curve above (8 K) and below (2 K) the superconducting transition of the JJ. The top-right inset shows the *I*–*V* curve near the zero-order minimum of *I*_c_(*μ*_0_*H*_⊥_). The solid lines correspond to our theoretical reproduction that takes both real-space magnetic texture (under an OOP magnetic field) and the *k*-space Weyl nodes into account (Supplementary Section 1). **e**, Magnified *I*_c_(*μ*_0_*H*_⊥_) plot around *μ*_0_*H*_⊥_ = 0 where asymmetric hysteretic *I*_c_(*μ*_0_*H*_⊥_) interference with the zero-order maximum-to-maximum offset Δ*μ*_0_*H*_⊥_ = 1.0 mT is evident. The error bars in **d** and **e** represent the standard deviation.

In this work, we demonstrate an antiferromagnetic analogue of the spin-triplet supercurrent spin-valve effect^4,13,14^ via the use of a single topological chiral antiferromagnet (AFM) Mn_3_Ge (refs. ^15–17^), which—with its lack of stray fields—can be highly advantageous for developing superconducting spintronic logic circuits^1^. The noteworthy aspect of Mn_3_Ge is that its non-collinear triangular antiferromagnetic spin arrangement^15–17^ in real space (Fig. 1a,b) and the fictitious magnetic fields derived from Berry curvature^18,19^ in *k* space, which are robust to low temperature *T* (refs. ^15–17,20^), facilitate the spin-mixing and spin-rotation processes^1–12^ required for singlet-to-triplet pair conversion. This enables, as shown in our recent experiment^20^, the proximity generation of long-range triplet supercurrents through the single chiral antiferromagnetic Josephson barrier.

## Chiral antiferromagnetic spin-triplet JJs

Our focus of the present study is on the Berry-curvature-driven fictitious fields^18,19^ that play an effective role in converting spin-unpolarized singlet Cooper pairs (*S* = 0) to form long-range triplets (*S* = 1) in the topological chiral AFM^20^. Note that how non-collinear six spins on a kagome bilayer^15–17^, constituting a cluster magnetic octupole, are arranged in real space (equivalently, how time-reversal symmetry is broken) determines the Berry curvature profile^18,19^ in *k* space. So, an external magnetic field *μ*_0_*H*_⊥_ applied perpendicular to the kagome plane tilts the overall antiferromagnetic spin arrangement to a certain extent to the field direction (Fig. 1a,b) and subsequently changes the associated Berry curvature^18^ around the Fermi energy and the resulting fictitious fields^19^, as theoretically calculated^21,22^. This offers, as shown below, a radically different approach to control the pair amplitude of triplets by applying an extremely small magnetic field (<2 mT). Our *k*-space Berry curvature approach is conceptually different from very recent works on controlling spin-triplet critical currents in a single FM with magnetic vortex^23^ and domain wall^24^, both of which focus on the real-space magnetic texturing across the FM Josephson barrier.

**Extended Data Fig. 1:**
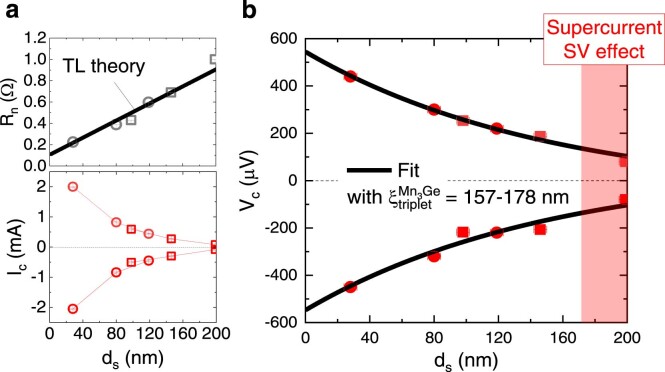
Long-range supercurrents and spin-valve effect in chiral antiferromagnetic JJs. **a**, Normal-state zero-bias resistance *R*_n_ (top) and Josephson critical current *I*_c_ (bottom) of the Nb/Mn_3_Ge/Nb JJs versus the barrier spacing *d*_s_. From *R*_n_(*d*_s_) in **a**, we extract the resistance-area product *r*_*i*_ of Nb/Mn_3_Ge interfaces to be 1.0–1.2 mΩ µm^2^ and the effective resistivity *ρ*_*ch*_ for the Mn_3_Ge(40 nm)/Ru(5 nm) track to be 25–27 µΩ cm, employing a standard transmission line (TL) theory^34^, $R_n = 2R_i + R_{ch} = \frac{{\rho _{ch}}}{{tw}}\left( {2l_t + d_s} \right)$. Here $R_i = \frac{{r_i}}{{wl_t}}$ and $l_t = \sqrt {\frac{{r_i}}{{\frac{{\rho _{ch}}}{t}}}}$ is the charge transfer length (13 nm). *t* and *w* is the thickness and width of the Mn_3_Ge/Ru track. **b**, Characteristic voltage *V*_c_ = *I*_c_*R*_n_ as a function of *d*_s_, from which we determine the decay length $\xi _{triplet}^{Mn_3Ge}$ of the Josephson supercurrents in the Mn_3_Ge barrier to be 157–178 nm using an exponential decay function^20^, $exp\left( { - \frac{{\xi _{triplet}^{Mn_3Ge}}}{{d_s}}} \right)$ (black curves). Note that for *d*_*s*_ ≥ $\xi _{triplet}^{Mn_3Ge}$, the *complete* supercurrent spin-valve effect appears (see Fig. 2d). All *I*_c_ values in **a**,**b** are obtained at a fixed temperature *T* = 2 K. Note that data with circle symbols in **a,b** are taken from Ref. ^20^. The error bars in **a** and **b** represent the standard deviation.

We carry out proof-of-concept experiments based on Nb/Mn_3_Ge/Nb lateral JJs^20^ (Fig. 1c). In particular, the edge-to-edge separation distance *d*_s_ of the adjacent superconducting Nb electrodes through an epitaxial thin film of the triangular chiral AFM Mn_3_Ge (Fig. 1a,b) is chosen to be comparable with or larger than the characteristic decay length $\xi _\mathrm{{triplet}}^{{\mathrm{Mn}}_3{\mathrm{Ge}}}$ = 157–178 nm (at *T* = 2 K; Extended Data Fig. 1) of spin-polarized triplet supercurrents^20^. Conventional wisdom is that the spin-unpolarized singlets (*S* = 0) are mostly exchange field filtered within a few nanometres^1–12^ and the surviving spin-polarized triplets (*S* = 1, *m*_*s*_ = ±1), which are immune to magnetic exchange fields^1–12^^,^^25^, finally mediate the long-range Josephson coupling. The mechanism for producing triplet Cooper pairs is a priori different in our case and we call this chiral antiferromagnetic spin-triplet JJs.

## Hysteretic out-of-kagome-plane magnetic-field interference patterns

**Extended Data Fig. 2:**
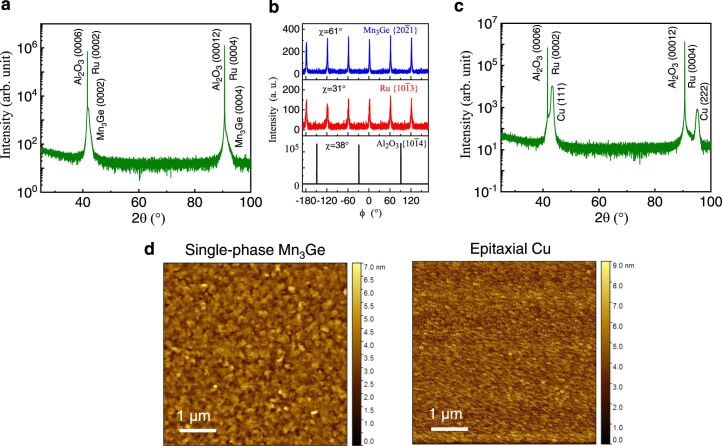
Structural analysis of the chiral non-collinear AFM Mn_3_Ge and the normal-metal Cu. **a**, Specular *θ*-2*θ* XRD pattern of single-crystalline Ru(0001) (black symbol) and single-phase Mn_3_Ge(0001)/Ru (red symbol) films grown on Al_2_O_3_(0001) substrates. **b**, Phi *ϕ* scan across the Al_2_O_3_ {$10\bar 14$}, Ru {$10\bar 13$}, and Mn_3_Ge {$20\bar 21$} reflections for the same film. **c**, Diffraction pattern of the epitaxial Cu film grown on Ru/Al_2_O_3_(0001) substrate. **d**, Atomic force micrographs of the single-phase Mn_3_Ge(0001) and epitaxial Cu films over 5 × 5 µm^2^ scan area (from left to right respectively). The root-mean-square roughness *R*_rms_ of the single-phase Mn_3_Ge (epitaxial Cu) is estimated to be 0.7 (0.6) nm. These data confirm that the single crystallinity and surface morphology of previous^20^ and current *D*0_19_-Mn_3_Ge(0001) films are almost identical.

Figure 1c,d shows the typical magnetic-field interference pattern of Josephson critical current *I*_c_(*μ*_0_*H*_⊥_) for the *d*_s_ = 199 nm JJ at *T* = 2 K. Here *μ*_0_*H*_⊥_ is applied along [0001] and thus perpendicular to the kagome plane of our single-phase hexagonal *D*0_19_-Mn_3_Ge(0001) layer (Fig. 1a,b and Methods). There exist two distinctively different features from the *I*_c_(*μ*_0_*H*_⊥_) interference pattern of our prior *d*_s_ ≤ 115 nm JJs^20^. First, the zero-order maximum of *I*_c_ appears 0.5–1.0 mT away from the zero field (*μ*_0_*H*_⊥_ = 0) and it is clearly hysteretic (Fig. 1e). As our single-phase *D*0_19_-Mn_3_Ge(0001) has a vanishingly small spontaneous magnetization (≤11 emu c.c.^–1^ at 2 K)^20^ in the kagome plane^15–17^^,20^, we ascribe this hysteretic *I*_c_(*μ*_0_*H*_⊥_) to the OOP-magnetic-field-modulated Berry curvature, as discussed later. Second, we obtain the characteristic *I*_c_(*μ*_0_*H*_⊥_) oscillation with clear minima for the *d*_s_ = 199 nm JJ (Fig. 1d), indicating the transverse uniformity of *I*_c_ across the whole Mn_3_Ge barrier and its coherent spatial quantum interference^26^. This improved magnetic-field interference in a longer JJ is probably due to the reduced effective edge roughness (several nanometres) of Nb electrodes relative to *d*_s_, given that the single crystallinity and surface morphology of previous^20^ and current *D*0_19_-Mn_3_Ge(0001) layers are not fundamentally different (Extended Data Fig. 2).

Our theory, considering a chirality-dependent phase $Qwd_\mathrm{s}\frac{{J\delta M}}{{\hbar v_\mathrm{F}}}\tau$ arising from the antiferromagnetic spin texture of Mn_3_Ge (Supplementary Section 1 provides the full details), anticipates the unique hysteretic Fraunhofer pattern and reproduces the overall *I*_c_(*μ*_0_*H*_⊥_) data (Fig. 1d, solid lines):

$$\begin{array}{l}I_\mathrm{c}\left( {\mu _0H_ \bot } \right) = I_0\left( {\left( {\mathop {\sum}\nolimits_\tau {\left( {1 + \tau \frac{{2\chi \gamma }}{{\gamma ^2 + \chi ^2}}} \right)} \sin \left( {d_\mathrm{s}\frac{{2JM_0}}{{\hbar v_\mathrm{F}}}\tau } \right){{{\mathrm{sinc}}}}\left( {\uppi \frac{{{{\varPhi }}_{\mathrm{JJ}}}}{{{{\varPhi }}_0}} + Qwd_\mathrm{s}\frac{{J\delta M}}{{\hbar v_\mathrm{F}}}\tau } \right)} \right)^2}\right.\\\left.{+ \left( {\mathop {\sum}\nolimits_\tau {\left( {1 + \tau \frac{{2\chi \gamma }}{{\gamma ^2 + \chi ^2}}} \right)} \cos \left( {d_\mathrm{s}\frac{{2JM_0}}{{\hbar v_\mathrm{F}}}\tau } \right){{{\mathrm{sinc}}}}\left( {\uppi \frac{{{{\varPhi }}_{\mathrm{JJ}}}}{{{{\varPhi }}_0}} + Qwd_\mathrm{s}\frac{{J\delta M}}{{\hbar v_\mathrm{F}}}\tau } \right)} \right)^2} \right)^{\frac{1}{2}}.\end{array}$$

Here *Q*, δ*M* and *J* are the inverse antiferromagnetic domain size, amplitude of the inhomogeneous part and exchange interaction of the antiferromagnetic spin texture of Mn_3_Ge, respectively; *τ* is the chirality index (±1); *γ* represents the transparency at the Nb/Mn_3_Ge interface; and *χ* describes the chirality dependence of the Mn_3_Ge barrier. Here *ħ* is the reduced Planck constant and *v*_F_ is the Fermi velocity of Mn_3_Ge. Also, ${{\varPhi }}_{\mathrm{JJ}} = \mu {_0}H_ \bot A_{\mathrm{JJ}}^{\mathrm{eff}}$ and $A_{\mathrm{JJ}}^{\mathrm{eff}}$ = (2*λ*_L_ + *d*_s_)*w* is the effective junction area of magnetic flux penetration (Fig. 1d, bottom inset), *λ*_L_ is the London penetration depth (130 nm at 2 K)^27^ of 50-nm-thick Nb electrodes, *w* is the width of the Mn_3_Ge barrier and ${{\varPhi }}_0 = \frac{h}{{2e}}$ = 2.07 × 10^−15^ T m^2^ is the magnetic flux quantum. From theoretical reproduction, we get *w* ≈ 1.1 µm, close to the actual width of our JJ (Fig. 1c), and $\left| {Qwd_{\mathrm{s}}\frac{{J\delta M}}{{\hbar v_{\mathrm{F}}}}\tau } \right|$ = 0.2 (Supplementary Section 2 provides a quantitative analysis).

## Spin-triplet supercurrent spin valves

**Fig. 2:**
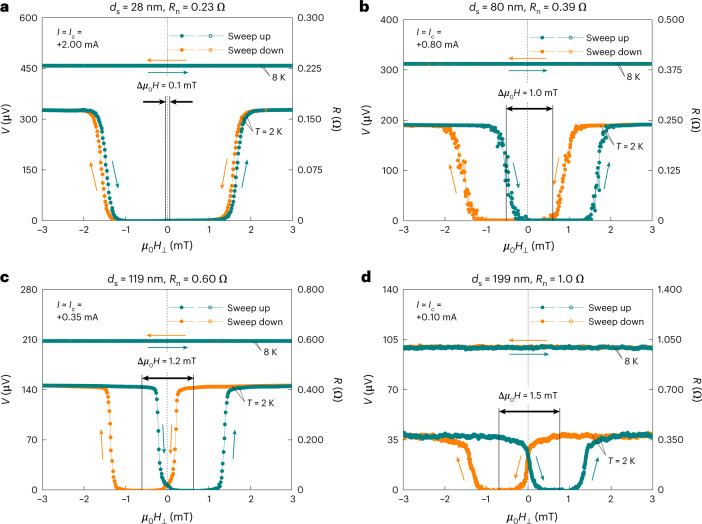
Supercurrent spin-valve effect in chiral antiferromagnetic spin-triplet JJs. **a**–**d**, Time-averaged voltage *V* as a function of external magnetic field *μ*_0_*H*_⊥_ for the d.c. current *I*-biased Nb/Mn_3_Ge/Nb JJs with different barrier spacing *d*_s_ values of 28 nm (**a**), 80 nm (**b**), 119 nm (**c**) and 199 nm (**d**), taken above (8 K) and below (2 K) the superconducting transition of the JJs. In these measurements, we apply fixed *I* that is similar to the zero-field Josephson critical current *I*_c_(*μ*_0_*H*_⊥_ = 0) of each JJ to straightforwardly visualize how the supercurrent spin-valve effect depends on *d*_s_. Note that *μ*_0_*H*_⊥_ (≤|3 mT|) is applied perpendicular to the kagome plane of the Mn_3_Ge barrier, and the JJ data in **a**–**c** are identical to what we used for our prior study^20^.

We now measure the time-averaged voltage *V* as a function of *μ*_0_*H*_⊥_ for the d.c. current *I*-biased JJs with *d*_s_ = 28–199 nm (Fig. 2a–d), from which, especially at *I* ≈ *I*_c_(*μ*_0_*H*_⊥_ = 0), one can straightforwardly see how the supercurrent spin-valve behaviour evolves as a function of *d*_s_. All the JJs in the superconducting state (*T* = 2 K) reveal asymmetric *V*(*μ*_0_*H*_⊥_) curves with respect to *μ*_0_*H*_⊥_ = 0 and their asymmetry is rigorously inverted when reversing the *μ*_0_*H*_⊥_ sweep direction. Note that since this asymmetric hysteretic behaviour disappears when the junctions are in the normal state (*T* = 8 K), it is necessarily connected to superconductivity induced in the chiral AFM leading to the Josephson supercurrent. With increasing *d*_s_, the centre-to-centre offset Δ*μ*_0_*H*_⊥_ between the sweep-up and sweep-down *V*(*μ*_0_*H*_⊥_) curves progressively broaden from 0.1 to 1.5 mT and the consequent asymmetric hysteresis becomes more evident. On reaching *d*_s_ = 199 nm (Fig. 2d), comparable with or larger than $\xi _{\mathrm{triplet}}^{{\mathrm{Mn}}_3{\mathrm{Ge}}}$ (157–178 nm; Extended Data Fig. 1) over which the proximity-created spin triplets can primarily mediate the long-range Josephson coupling^1–12^, the complete antiferromagnetic analogue of the spin-triplet supercurrent spin valve^4,13,14^ is established in our chiral antiferromagnetic JJ. The applied *μ*_0_*H*_⊥_ ≤ 2.0 mT here to turn the Josephson supercurrent on and off (Fig. 2d) is intriguingly one order of magnitude smaller than that typically required for ferromagnetic spin-triplet JJs^13,14^, where one should apply a magnetic field larger than the coercive field of the free spin-rotator FM_2_ (for example, a few tens of millitesla even for soft FM Ni_8_Fe_2_)^13^ to change its magnetization direction relative to the pinned spin-mixer FM_1_, providing a beneficial route for controlling the pair amplitude of the spin triplets.

## Supercurrent spin valve and 0-to-π phase shift in a d.c. SQUID

**Fig. 3:**
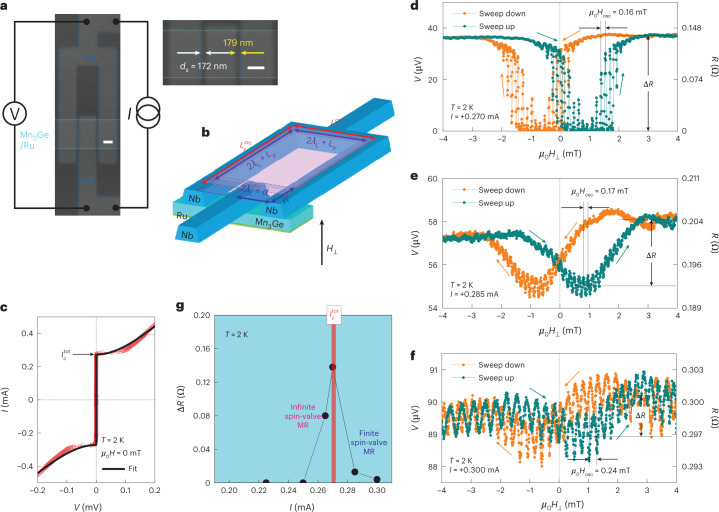
Spin-triplet supercurrent spin valve implemented in Mn_3_Ge JJ-based SQUID. **a**,**b**, Scanning electron micrographs (**a**) and measurement scheme (**b**) of the fabricated d.c. SQUID, which contains two Nb/Mn_3_Ge/Nb JJs with barrier spacing *d*_s_ = 172 and 179 nm (≥$\xi _{\mathrm{triplet}}^{{\mathrm{Mn}}_3{\mathrm{Ge}}}$) that are laterally connected through the single layer of *D*0_19_-Mn_3_Ge(0001). Scale bar, 0.5 µm (**a**). Note that if the width of the SQUID track is much larger than the London penetration depth *λ*_L_, flux focusing effectively widens the SQUID area to be $L_x^{\mathrm{ctc}}L_y^{\mathrm{ctc}}$, where $L_{x,y}^{\mathrm{ctc}}$ is the centre-to-centre spacing between the tracks defining the two opposite sides of the SQUID. Zero-field *I*–*V* curve of the Mn_3_Ge JJ-based SQUID at *T* = 2 K. **d**–**f**, Time-averaged voltage *V* as a function of perpendicular magnetic field *μ*_0_*H*_⊥_ for the *I*-biased SQUID, taken at *T* = 2 K. From the periodic $V(\mu _0H_ \bot ,\;I \ge I_\mathrm{c}^{\mathrm{tot}})$ modulation in **d**–**f**, we find a characteristic period of *μ*_0_*H*_osc_ = 0.16‒0.24 mT (Extended Data Fig. 3). **g**, Spin-valve amplitude Δ*R* = *R*_high_ – *R*_low_, implemented in the SQUID *V*(*μ*_0_*H*_⊥_) oscillation versus *I*. Note that for $I \lesssim I_\mathrm{c}^{\mathrm{tot}}(\mu _0H_ \bot = 0)$, we achieve an infinite spin-valve magnetoresistance ${{{\mathrm{MR}}}} = \frac{{\Delta R}}{{R_{\mathrm{low}}}} \to \infty$ by definition, indicative of rigorous switching between quasiparticle currents and supercurrents.

By taking advantage of this low-field spin-triplet supercurrent spin valve, we next fabricate a direct-current superconducting quantum interference device (d.c. SQUID) to showcase its potential as an on-chip local probe^28^ of out-of-kagome-plane magnetic moments of chiral AFMs with high sensitivity. Note that because the active superconducting loop of our SQUID (Fig. 3a,b and Methods) contains two chiral antiferromagnetic spin-triplet JJs of *d*_s_ = 172 and 179 nm (≥$\xi _{\mathrm{triplet}}^{{\mathrm{Mn}}_3{\mathrm{Ge}}}$) that are laterally connected through the single layer of *D*0_19_-Mn_3_Ge(0001), the SQUID action of this device is available only when the superconducting Nb electrodes are Josephson coupled via spin-triplet Cooper pairs^24,29^. From the zero-field current–voltage (*I*–*V*) curve of the fabricated SQUID (Fig. 3c), we find that the total critical current $I_\mathrm{c}^{\mathrm{tot}}$ is approximately twice the *I*_c_ value of a single JJ with similar *d*_s_ (Extended Data Fig. 1). This matches the standard theory^26^ of a d.c. SQUID comprising two overdamped JJs^20,26^ with a low resistance–capacitance product, that is,

$$I_\mathrm{c}^{\mathrm{tot}}\left( {\mu _0H_ \bot } \right) = \sqrt {\left( {I_{\mathrm{c}1} - I_{\mathrm{c}2}} \right)^2 + 4I_{\mathrm{c}1}I_{\mathrm{c}2}\left( {\cos \left( {\uppi \frac{{{{\varPhi }}_{\mathrm{SQUID}}}}{{{{\varPhi }}_0}} + \left( {\frac{{\varphi _1 + \varphi _2}}{2}} \right)} \right)} \right)^2}.$$

Here we assume a small self-inductance of the SQUID loop for simplicity and consider the low-field regime (*Φ*_JJ_ ≪ *Φ*_0_) such that the $I_\mathrm{c}^{\mathrm{tot}}\left( {\mu _0H_ \bot } \right)$ curve mostly reflects the SQUID characteristics. Here *I*_c1_ (*I*_c2_) and *φ*_1_ (*φ*_2_) are the zero-field Josephson critical current and intrinsic phase difference^30^ for the first (second) JJ of the SQUID, respectively. Also, $\varPhi _{\mathrm{SQUID}} = \mu _0H_ \bot A_{\mathrm{SQUID}}^{\mathrm{eff}}$ is the magnetic flux threading the SQUID loop given by *μ*_0_*H*_⊥_ and $A_{\mathrm{SQUID}}^{\mathrm{eff}}$ = (2*λ*_L_ + *L*_*x*_)(2*λ*_L_ + *L*_*y*_) is the effective SQUID area (Fig. 3b). Note that for *μ*_0_*H*_⊥_ = 0, $I_\mathrm{c}^{\mathrm{tot}} = I_{\mathrm{c}1} + I_{\mathrm{c}2}$.

Most importantly, the low-field supercurrent spin-valve functionality, that is, the active modulation of Josephson coupling strength as well as the ground-state phase difference, is successfully implemented in the SQUID oscillation (Fig. 3d–f). To the best of our knowledge, only a recent work utilizing multiple FMs has succeeded in the controllable switching between 0- and π-phase states of the ferromagnetic spin-triplet JJs embedded in a d.c. SQUID^29^. As summarized in Fig. 3g, the implemented spin-valve amplitude Δ*R* = *R*_high_ – *R*_low_ increases with increasing *I* and achieves the maximum value at $I \approx I_\mathrm{c}^{\mathrm{tot}}\left( {\mu _0H_ \bot = 0} \right)$, followed by a strong drop for larger *I*. Especially for $I \lesssim I_\mathrm{c}^{\mathrm{tot}}\left( {\mu _0H_ \bot = 0} \right)$, we obtain an infinite spin-valve magnetoresistance ${{{\mathrm{MR}}}} = \frac{{\Delta R}}{{R_{\mathrm{low}}}} \to \infty$ by definition with rigorous switching between quasiparticle currents and supercurrents (Fig. 3d,g). This demonstration of the SQUID spin-valve oscillation can be used to devise a phase-resolved and magnetization-component-specific detector of antiferromagnetic domain walls, which remains a major challenge in the research field of AFM spintronics^28^.

**Extended Data Fig. 3:**
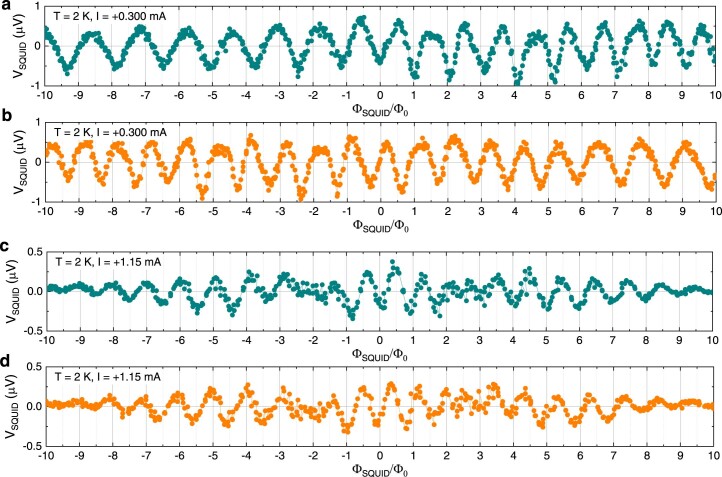
Magnetic-flux-to-voltage conversion in Mn_3_Ge JJ- or Cu JJ-based SQUID. SQUID voltage *V*_*SQUID*_ oscillation as a function of normalized magnetic flux ${\it{\Phi }}_{SQUID}/{\it{\Phi }}_0$ for the *I* = +0.30 mA-biased Mn_3_Ge JJ-based SQUID, taken at *T* = 2 K when sweeping *μ*_0_*H*_⊥_ up (**a**) and down (**b**). Note that we set $A_{SQUID}^{eff}$ = 9 µm and subtract background low-order polynomial and asymmetric voltage signals are from Fig. 3f for clarity. **c**,**d**, Data equivalent to **a**,**b** but for the *I* = +1.15 mA-biased Cu JJ-based SQUID. Here $A_{SQUID}^{eff}$ = 12 µm is set and background low-order polynomial voltage signals are subtracted from Fig. 4f. Unlike the Cu JJ-based SQUID, there exists a finite phase shift *φ*_1_ + *φ*_2_ ≈ *π* in the sweep-up and sweep-down $V_{SQUID}\left( {{\it{\Phi }}_{SQUID}/{\it{\Phi }}_0} \right)$ data of the Mn_3_Ge JJ-based SQUID. This implies that the rotational chirality^29^ and ground-state phase difference of our chiral antiferromagnetic spin-triplet JJs seems to be controlled by *μ*_0_*H* (see main text for details).

For the *I*-biased SQUID of two overdamped JJs in the limit of small self-inductance and in the low-field limit (*Φ*_JJ_ ≪ *Φ*_0_), the conversion of a magnetic flux into *V* modulation can be approximated by^26^

$$V\left( {\mu _0H_ \bot ,\;I} \right) = \frac{{R_{\mathrm{n}1}R_{\mathrm{n}2}}}{{R_{\mathrm{n}1} + R_{\mathrm{n}2}}}\sqrt {(I)^2 - \left( {I_\mathrm{c}^{\mathrm{tot}}\cos\left( {\uppi \frac{{{{\varPhi }}_{\mathrm{SQUID}}}}{{{{\varPhi }}_0}} + \left( {\frac{{\varphi _1 + \varphi _2}}{2}} \right)} \right)} \right)^2},$$

where *R*_n1_ (*R*_n2_) is the normal-state zero-bias resistance of the first (second) JJ. From the measured $V\left( {\mu _0H_ \bot ,\;I \ge I_\mathrm{c}^{\mathrm{tot}}} \right)$ oscillation with a period of *μ*_0_*H*_osc_ = 0.16‒0.24 mT (Fig. 3d–f and Extended Data Fig. 3) and using the relationship $\mu _0H_{\mathrm{osc}} = \frac{{{{\varPhi }}_0}}{{A_{\mathrm{SQUID}}^{\mathrm{eff}}}}$, we obtain $A_{\mathrm{SQUID}}^{\mathrm{eff}}$ = 9‒13 μm^2^. This value is 2‒3 times larger than the geometrical area of the SQUID loop ((2*λ*_L_ + *L*_*x*_)(2*λ*_L_ + *L*_*y*_) = 4.1 μm^2^), which we attribute to a flux-focusing effect. Note that if the width of the SQUID loop is much larger than *λ*_L_, the flux-focusing effect comes into play and effectively widens the SQUID area to be $L_x^{\mathrm{ctc}}L_y^{\mathrm{ctc}}$ ≈ 11 μm^2^ (Fig. 3b), indicating the reliable performance of our SQUID. Here $L_{x,y}^{\mathrm{ctc}}$ is the centre-to-centre spacing between the tracks defining the two opposite sides of the SQUID.

**Fig. 4:**
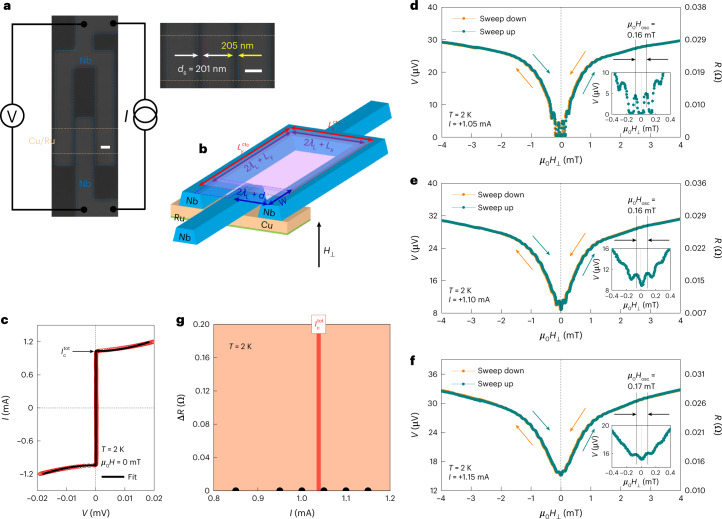
Absence of supercurrent spin-valve effect in Cu JJ-based SQUID. **a**–**g**, Data equivalent to Fig. 3a–g but for the d.c. SQUID composed of two Nb/Cu/Nb JJs with longer *d*_s_ = 201 and 205 nm, in which the Cu spacer is epitaxial (Extended Data Fig. 2). Scale bar, 0.5 µm (**a**). Note that contrary to the Mn_3_Ge JJ-based SQUID, no asymmetric hysteretic response of time-averaged voltage *V* to perpendicular magnetic field *μ*_0_*H*_⊥_(≤|3 mT|) is detected in the Cu-JJ-based SQUID (**g**). From the periodic $V\left( {\mu _0H_ \bot ,\;I \ge I_\mathrm{c}^{\mathrm{tot}}} \right)$ modulation (insets of **d**–**f**), we find a characteristic period of *μ*_0_*H*_osc_ = 0.16‒0.17 mT (Extended Data Fig. 3).

**Extended Data Fig. 4:**
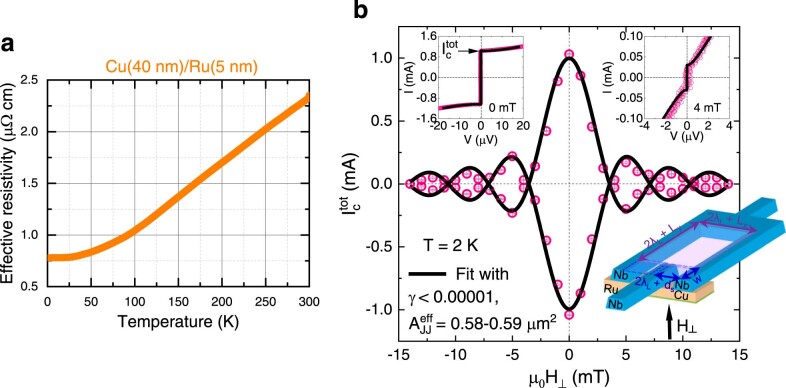
Magnetic-field interference pattern of the Cu JJ-based SQUID. **a**, Estimated electrical resistivity versus temperature *T* plot for the Cu(40 nm)/Ru(5 nm) reference Hall-bar device. **b**, Total critical current $I_c^{tot}$ versus *μ*_0_*H*_⊥_ plot for the Cu JJ-based SQUID, taken at *T* = 2 K. Here *μ*_0_*H*_⊥_ is applied perpendicular to the interface plane of Nb electrodes, as illustrated in the bottom inset. The top left inset displays the zero-field current-voltage *I*-*V* curve at 2 K whereas the top right inset shows the *I*-*V* curve near the zero-order minimum of $I_c^{tot}$(*μ*_0_*H*_⊥_). To focus on a full modulation of $I_c^{tot}$ caused by the magnetic-field quantum interference of two Nb/Cu/Nb lateral JJs, we measure the $I_c^{tot}$(*μ*_0_*H*_⊥_) curves at a rather large interval (1 mT) of *μ*_0_*H*_⊥_. The black solid lines in **b** are fitting curves^20^ to determine the supercurrent non-uniformity γ and the effective junction area A^eff^_JJ_. The error bars in **b** represent the standard deviation.

**Extended Data Fig. 5:**
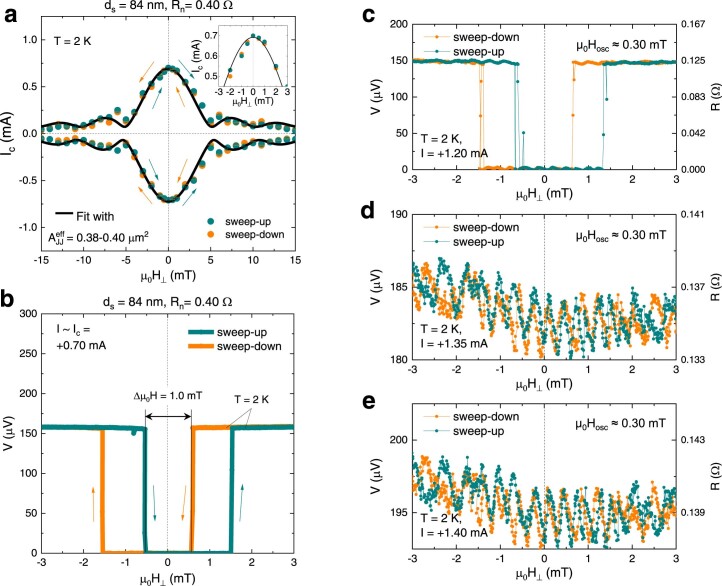
No supercurrent spin-valve effect and 0-to-π phase shift detectable in the ${\it{d}}_{\it{s}} < {\it{\xi }}_{{\it{triplet}}}^{{\it{Mn}}_3{\it{Ge}}}$ Mn_3_Ge JJ-based SQUID. **a**, Typical magnetic-field interference pattern *I*_*c*_(*μ*_0_*H*_⊥_) of the *d*_s_ = 84 nm (<$\xi _{triplet}^{Mn_3Ge}$ = 157–178 nm) JJ. The inset displays the magnified plot around the zero field *μ*_0_*H*_⊥_ = 0. **b**, Time-averaged voltage *V* as a function of external magnetic field *μ*_0_*H*_⊥_ for the dc current *I*-biased *d*_s_ = 84 nm JJ, taken at 2 K. In this measurement, *μ*_0_*H*_⊥_ (≤|3 mT|) is applied perpendicular to the Kagome plane of the Mn_3_Ge barrier. **c**-**e**, Time-averaged voltage *V* as a function of perpendicular magnetic field *μ*_0_*H*_⊥_ for the *I*-biased SQUID, taken at *T* = 2 K. From the periodic $V\left( {\mu _0H_ \bot ,\;I \ge I_c^{tot}} \right)$ modulation in **c**-**e**, we find a characteristic period of *μ*_0_*H*_*osc*_ ≈ 0.30 mT. None of the supercurrent spin-valve behaviour and the 0-to-π phase shift as a function of *μ*_0_*H*_⊥_ clearly emerge in the $d_s \approx 80\;nm\;\left( { < \xi _{triplet}^{Mn_3Ge}} \right)$ Mn_3_Ge JJ-based SQUID, which are well consistent with our theoretical modelling [Eq. (S28)]. The error bars in **a** represent the standard deviation.

Interestingly, from a comparison of the sweep-up and sweep-down *V*_SQUID_(*μ*_0_*H*_⊥_) data (Fig. 3f and Extended Data Fig. 4), a finite phase shift of *φ*_1_ + *φ*_2_ ≈ π is evident, which does not exist in a normal-metal Cu-JJ-based SQUID (Fig. 4 and Extended Data Fig. 4). In ferromagnetic spin-triplet JJs^29^, whether the JJ will be a 0 junction or a π junction depends on the sum of the rotational chirality from left spin-mixer FM_1_ to central spin-rotator FM_2_ and that from central spin-rotator FM_2_ to right spin-mixer FM_3_. If the JJ has the same rotational chirality across the entire FM_1_/FM_2_/FM_3_ Josephson barrier, then the junction will be a 0 junction, whereas if it has the opposite rotational chirality across the FM_1_/FM_2_/FM_3_ barrier, then the junction will be a π junction. This suggests that the OOP rotational chirality and ground-state phase difference of our chiral antiferromagnetic spin-triplet JJs seem to be controlled by external OOP magnetic fields. In fact, our theoretical modelling (Methods and Supplementary Section 1) assures that both Josephson critical current and phase shift crucially depend on the chiral antiferromagnetic spin structure (or spin textures in the chiral AFM), which can change when the OOP magnetic field is swept. As presented by equation (23) in Supplementary Section 1, our theory predicts that for $d_{\mathrm{s}}\frac{{2JM_0}}{{\hbar v_{\mathrm{F}}}}\tau > \uppi /2$, the JJ can transition to a π junction from a 0 junction. Given our theory (equation (28) in Supplementary Section 1) that $\frac{{2J\delta M}}{{\hbar v_{\mathrm{F}}}}$ is the inverse decay length of triplet supercurrents $\left({\xi _{\mathrm{triplet}}^{{\mathrm{Mn}}_3{\mathrm{Ge}}}}\right)^{ - 1}$ and δ*M* is in the same order as *M*_0_, we theoretically expect the 0-to-π transition appearing for $d_{\mathrm{s}} > \frac{\uppi }{2}\left( {\frac{{\hbar v_{\mathrm{F}}}}{{2JM_0}}} \right)\approx \frac{\uppi }{2}\xi _{\mathrm{triplet}}^{{\mathrm{Mn}}_3{\mathrm{Ge}}}$ ≈ 200 nm (whose value is taken from Extended Data Fig. 1). This agrees with what we observe (Fig. 3f and Extended Data Fig. 4). We also emphasize that for *d*_s_ ≈ 80 nm(<$\xi _{\mathrm{triplet}}^{{\mathrm{Mn}}_3{\mathrm{Ge}}}$) Mn_3_Ge JJ-based SQUID (Extended Data Fig. 5), none of the supercurrent spin-valve behaviour and the 0-to-π phase shift as a function of *μ*_0_*H*_⊥_ clearly emerge, which is again consistent with our theoretical prediction (equation (28) in Supplementary Section 1).

By substituting the chiral AFM Mn_3_Ge with normal-metal Cu (Fig. 4a,b), we also perform a control experiment to check a possible contribution of OOP Abrikosov vortex nucleation under *μ*_0_*H*_⊥_. The Cu-JJ-based SQUID reveals higher $I_\mathrm{c}^{\mathrm{tot}}\left( {\mu _0H_ \bot = 0} \right)$ even with larger *d*_s_ (201 and 205 nm; Fig. 4c) than the Mn_3_Ge JJ-based SQUID, as would be expected for the long singlet superconducting proximity effect (a few hundred nanometres) in the highly conductive normal-metal Cu barrier^31^. Its well-defined $I_\mathrm{c}^{\mathrm{tot}}(\mu _0H_ \bot )$ interference pattern in modest fields further supports a good Josephson property (Extended Data Fig. 4). When biasing $I \ge I_\mathrm{c}^{\mathrm{tot}}\left( {\mu _0H_ \bot = 0} \right)$ to the Cu-JJ-based SQUID (Fig. 4d–f), we do not observe any asymmetric hysteretic behaviour in the *V*(*μ*_0_*H*_⊥_) curves (Fig. 4g) but do measure the clear *V*(*μ*_0_*H*_⊥_) oscillation of *μ*_0_*H*_osc_ = 0.16‒0.17 mT (Extended Data Fig. 3). This result indicates that OOP Abrikosov vortices are unlikely to be the source of the found supercurrent spin-valve behaviour.

## Conclusions

**Extended Data Fig. 6:**
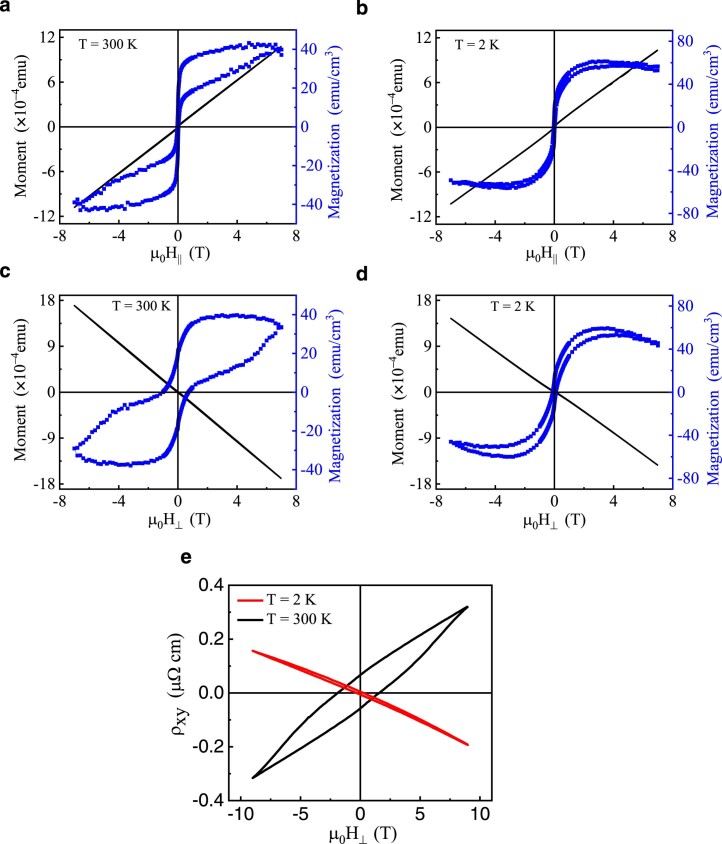
Magnetic properties and AHE of the chiral non-collinear AFM Mn_3_Ge. **a**,**b**, Typical magnetic hysteresis *M*(*μ*_0_*H*_||_) curves of the single-phase 40 nm Mn_3_Ge(0001) film at 300 K and 2 K, where *μ*_0_*H*_||_ is applied parallel to the Kagome *ab* plane. Black represents the raw measured data and blue represents the corrected data after subtracting the paramagnetic contribution. These data are taken from Ref. ^20^. **c**,**d**, Data equivalent to **a**,**b** but for the *M*(*μ*_0_H_⊥_) curves, where *μ*_0_*H*_⊥_ is applied perpendicular to the Kagome *ab* plane. **e**, Representative Hall resistivity versus magnetic field *R*-*H* curves of the single-phase Mn_3_Ge(0001) at 2 and 300 K. These data, taken from Ref. ^20^, suggest that there exists vanishingly small but finite OOP canted magnetization of our *D*0_19_-Mn_3_Ge(0001) film, which is in line with the case of bulk Mn_3_Ge^15^.

Recent theories^21,22^ have suggested that the out-of-kagome-plane overall tilting of the triangular non-collinear AFM spin arrangement by a few degrees in Mn_3_X (X = Ir, Sn, Ge) indeed visibly changes the *k*-space Berry curvature near the Fermi energy and the associated anomalous Hall response. In addition, vanishingly small but finite OOP canted magnetization of our *D*0_19_-Mn_3_Ge(0001) film (Extended Data Fig. 6) enables the hysteretic behaviour of Josephson supercurrents as a function of *μ*_0_*H*_⊥_. These further support our claim that magnetic-field-controlled triplet pairing states through the Berry curvature modification can lead to the spin-triplet supercurrent spin-valve effect even in a single topological chiral AFM. In fact, our theory, which takes both real-space magnetic texture (under an OOP magnetic field) and *k*-space Weyl nodes into account (Supplementary Section 1), reproduces the observed hysteric Fraunhofer pattern (Fig. 1d). Although the theory has to be further developed, especially regarding full boundary conditions for quasiclassical Green’s functions and their chirality dependence, our present model reasonably captures the physics behind our experimental findings (that is, hysteresis in the Fraunhofer pattern and 0-to-π phase shift in the SQUID data). How microscopic details of antiferromagnetic spin texturing and out-of-kagome-plane titling affect the chirality-dependent phase also need to be systematically studied in the future. We believe that our result facilitates a better understanding of the role of the Berry curvature in singlet-to-triplet pair conversion, inspires future theoretical studies on the interplay of Berry curvature and spin-triplet pairing in a chiral non-collinear AFM in more detail and provides a radical route for controlling the triplet-pair amplitude by an extremely small magnetic field—an essential prerequisite for logic circuit^1^ or AFM domain-wall sensor^28^ applications of spin-triplet supercurrents.

## Methods

### Sample growth and characterization

Single-phase hexagonal *D*0_19_-Mn_3_Ge(0001) (ref. ^20^) and Cu thin films were epitaxially grown on a Ru-buffered Al_2_O_3_(0001) substrate by d.c. magnetron sputtering in an ultrahigh-vacuum system with a base pressure of 1 × 10^−9^ torr. A 5-nm-thick Ru buffer layer was first sputtered at 450 °C with a sputtering power of 15 W and Ar pressure of 3 mtorr. Subsequently, Mn and Ge were co-deposited from elemental sputter targets on the Ru(0001) buffer layer at 500 °C and Ar pressure of 3 mtorr, where the sputter powers of 31 and 10 W for Mn and Ge, respectively, were used. Note that these growth conditions are essentially the same as those used for our recent study^20^. On the other hand, the epitaxial Cu layer (Extended Data Fig. 2) was sputtered at 27 °C with a sputter power of 15 W and Ar pressure of 3 mtorr. All these single-phase hexagonal *D*0_19_-Mn_3_Ge(0001) (ref. ^20^) and Cu epitaxial films were capped with a 1-nm-thick AlO_*x*_ layer to prevent oxidation. We performed the structural and magnetic characterizations of the prepared thin films using X-ray diffraction and SQUID vibrating-sample magnetometer, respectively. To investigate the Berry-curvature-driven anomalous Hall effect^15–17,20^, we also carried out magnetotransport measurements on the unpatterned *D*0_19_-Mn_3_Ge(0001) film in the van der Pauw geometry.

### Lithography patterning and device fabrication

As the fabrication procedure of lateral Nb/Mn_3_Ge/Nb JJs (Fig. 1b) was previously discussed^20^, here we only describe the fabrication steps for the d.c. SQUID (Figs. 3a and 4a). A central track of either *D*0_19_-Mn_3_Ge/Ru (Fig. 3a) or Cu/Ru epitaxial layers (Fig. 4a) with lateral dimensions of 1.5 × 50.0 μm^2^ was first defined using optical lithography and Ar-ion-beam etching, and then Au (80 nm)/Ru (2 nm) electrical leads and bonding pads were deposited by Ar-ion-beam sputtering. We subsequently defined the SQUID loop with an inner area of 1.0 × 3.0 μm^2^ (Figs. 3a and 4a), in which two constituent JJs were formed on top of the Mn_3_Ge/Ru (Fig. 3a) or Cu/Ru (Fig. 4a) track via electron-beam lithography and lift-off steps. The 50-nm-thick Nb electrodes were grown by Ar-ion-beam sputtering at a pressure of 1.5 × 10^–4^ mbar and the two constituent JJs are edge-to-edge separated by ≥$\xi _{\mathrm{triplet}}^{{\mathrm{Mn}}_3{\mathrm{Ge}}}$ (157–178 nm; Extended Data Fig. 1). For direct metallic electrical contacts, the Al_2_O_3_ capping layer and Au surface were etched away by an Ar-ion beam before sputtering the Nb electrodes.

### Low-temperature transport measurement

We measured the *I*–*V* curves of the fabricated JJs (Fig. 1d) and d.c. SQUID (Figs. 3c and 4c) with a four-probe configuration in a Quantum Design physical property measurement system using a Keithley 6221 current source and Keithley 2182A nanovoltmeter. The Josephson critical current *I*_c_ and normal-state zero-bias resistance *R*_n_ of each JJ (Extended Data Fig. 1) were determined by fitting the measured *I*–*V* curves with the standard formula for overdamped junctions^26^, namely, $V(I) = \frac{I}{{\left| I \right|}}R_\mathrm{n}\sqrt {I^2 - I_\mathrm{c}^2}$. We obtained the magnetic-field interference pattern *I*_c_(*μ*_0_*H*) (Fig. 1d,e and Extended Data Fig. 4) by repeating the *I*–*V* measurements at *T* = 2 K with varying magnetic fields *μ*_0_*H*_⊥_, applied perpendicular to the kagome plane of *D*0_19_-Mn_3_Ge(0001), from negative to positive values, and vice versa. We subsequently measured the *V*(*μ*_0_*H*_⊥_) curves for the *I*-biased JJs (Fig. 2a–d) and d.c. SQUID (Figs. 3d–g and 4d–g) by sweeping *μ*_0_*H*_⊥_ up and down.

### Quasiclassical theory of superconducting proximity effect and spin-triplet supercurrent spin-valve effect

As presented in Supplementary Section 1, we developed the quasiclassical theory of the superconducting proximity effect in a conventional AFM and chiral AFM. We derived the equations describing the propagation of superconducting correlations in the diffusive limit—Usadel equations—that are relevant to the devices studied experimentally here. We found that all the superconducting correlations of spin-unpolarized singlets (*S* = 0) and spin-zero (*S* = 1, *m*_*s*_ = 0) and spin-polarized (*S* = 1, *m*_*s*_ = ±1) triplets are strongly damped by exchange spin-splitting fields in the conventional AFM, leading to a short-ranged proximity effect^25,32,33^. However, in case of chiral AFM, spin-momentum locking along with the Weyl node structure turned out to cause a distinctively different superconducting proximity effect. The spin-momentum locking implies that spin texturing in the chiral AFM plays the role of a vector potential, thereby phase shifting the superconducting order parameter and inducing a *φ*-junction (or π-junction) behaviour, which is controlled by modulating the chiral antiferromagnetic spin texturing. Note also that the Weyl node structure (directly relevant to the Berry curvature) imposes the existence of spin-triplet correlations inside the chiral AFM (equation (16) in Supplementary Section 1). These correlations are expected to propagate over a long distance (equation (17) in Supplementary Section 1).

When the OOP magnetic field is applied and swept, the amplitude *I*_Chiral_ and phase *φ*_0,Chiral_ of the Josephson triplet supercurrent through the *d*_s_ > 150 nm Mn_3_Ge barrier can both visibly change because these values scale directly with *d*_s_ and are determined by how the antiferromagnetic spin texture of Mn_3_Ge is configured (Supplementary Section 1). This is the theoretical insight that reasonably explains all the experimental findings of the present paper. Full details of our quasiclassical theory, which reproduces the hysteretic Fraunhofer pattern (Fig. 1d) and explains the 0-to-π phase shift in the SQUID data (Extended Data Fig. 3a,b), can be found in equations (24)–(28) in Supplementary Section 1.

## Online content

Any methods, additional references, Nature Portfolio reporting summaries, source data, extended data, supplementary information, acknowledgements, peer review information; details of author contributions and competing interests; and statements of data and code availability are available at 10.1038/s41565-023-01336-z.

## Supplementary information

Supplementary Information Supplementary Sections 1–3, Fig. 1 and refs. 1–14.

## Data Availability

The data used in this Article are available from the corresponding authors upon reasonable request.

## References

[1] Linder J, Robinson JWA (2015). Superconducting spintronics. Nat. Phys..

[2] Eschrig M (2015). Spin-polarized supercurrents for spintronics: a review of current progress. Rep. Prog. Phys..

[3] Birge NO (2018). Spin-triplet supercurrents in Josephson junctions containing strong ferromagnetic materials. Philos. Trans. R. Soc. A.

[4] Houzet M, Buzdin AI (2007). Long range triplet Josephson effect through a ferromagnetic trilayer. Phys. Rev. B.

[5] Bergeret FS, Volkov AF, Efetov KB (2001). Long-range proximity effects in superconductor-ferromagnet structures. Phys. Rev. Lett..

[6] Cottet A (2011). Inducing odd-frequency triplet superconducting correlations in a normal metal. Phys. Rev. Lett..

[7] Bergeret FS, Tokatly IV (2014). Spin–orbit coupling as a source of long-range triplet proximity effect in superconductor–ferromagnet hybrid structures. Phys. Rev. B.

[8] Robinson JWA, Witt JDS, Blamire MG (2010). Controlled injection of spin-triplet supercurrents into a strong ferromagnet. Science.

[9] Khaire TS, Khasawneh MA, Pratt WP, Birge NO (2010). Observation of spin-triplet superconductivity in Co-based Josephson junctions. Phys. Rev. Lett..

[10] Keizer RS (2006). A spin triplet supercurrent through the half-metallic ferromagnet CrO_2_. Nature.

[11] Jeon K-R (2018). Enhanced spin pumping into superconductors provides evidence for superconducting pure spin currents. Nat. Mater..

[12] Banerjee N (2018). Controlling the superconducting transition by spin-orbit coupling. Phys. Rev. B.

[13] Banerjee N, Robinson JWA, Blamire MG (2014). Reversible control of spin-polarized supercurrents in ferromagnetic Josephson junctions. Nat. Commun..

[14] Martinez WM, Pratt WP, Birge NO (2016). Amplitude control of the spin-triplet supercurrent in S/F/S Josephson junctions. Phys. Rev. Lett..

[15] Nayak AK (2016). Large anomalous Hall effect driven by a nonvanishing Berry curvature in the noncolinear antiferromagnet Mn_3_Ge. Sci. Adv..

[16] Kiyohara N, Tomita T, Nakatsuji S (2016). Giant anomalous Hall effect in the chiral antiferromagnet Mn_3_Ge. Phys. Rev. Appl..

[17] Soh J-R (2020). Ground-state magnetic structure of Mn_3_Ge. Phys. Rev. B.

[18] Xiao D, Chang M-C, Niu Q (2010). Berry phase effects on electronic properties. Rev. Mod. Phys..

[19] Chen T (2021). Anomalous transport due to Weyl fermions in the chiral antiferromagnets Mn_3_X, X = Sn, Ge. Nat. Commun..

[20] Jeon K-R (2021). Long-range supercurrents through a chiral non-collinear antiferromagnet in lateral Josephson junctions. Nat. Mater..

[21] Chen H, Niu Q, MacDonald AH (2014). Anomalous Hall effect arising from noncollinear antiferromagnetism. Phys. Rev. Lett..

[22] Busch O, Göbel B, Mertig I (2020). Microscopic origin of the anomalous Hall effect in noncollinear kagome magnets. Phys. Rev. Research.

[23] Fermin R (2022). Superconducting triplet rim currents in a spin-textured ferromagnetic disk. Nano Lett..

[24] Bhatia E (2021). Nanoscale domain wall engineered spin-triplet Josephson junctions and SQUID. Nano Lett..

[25] Komori S (2021). Spin-orbit coupling suppression and singlet-state blocking of spin-triplet Cooper pairs. Sci. Adv..

[26] Barone, A. & Paterno, G. *Physics and Applications of the Josephson Effect* 2nd edn (John Wiley & Sons, 1982).

[27] Gubin AI, Il’in KS, Vitusevich SA (2005). Dependence of magnetic penetration depth on the thickness of superconducting Nb thin films. Phys. Rev. B.

[28] Jungwirth T, Marti X, Wadley P, Wunderlich J (2016). Antiferromagnetic spintronics. Nat. Nanotechnol..

[29] Glick JA (2018). Phase control in a spin-triplet SQUID. Sci. Adv..

[30] Guichard W (2003). Phase sensitive experiments in ferromagnetic-based Josephson junctions. Phys. Rev. Lett..

[31] Blum Y, Tsukernik A, Karpovski M, Palevski A (2002). Oscillations of the superconducting critical current in Nb-Cu-Ni-Cu-Nb junctions. Phys. Rev. Lett..

[32] Bell C (2003). Proximity and Josephson effects in superconductor/antiferromagnetic Nb/γ-Fe_50_Mn_50_ heterostructures. Phys. Rev. B.

[33] Weides M, Disch M, Kohlstedt H, Bürgler DE (2009). Observation of Josephson coupling through an interlayer of antiferromagnetically ordered chromium. Phys. Rev. B.

[34] Schroder, D. K. *Semiconductor Material and Device Characterization* 2nd edn (Wiley-Blackwell, 1998).

