# Supplementary material for: Chiral antiferromagnetic Josephson junctions as spin-triplet supercurrent spin valves and d.c. SQUIDs
Source: Nat Nanotechnol. 2023 Mar 30;18(7):747–53. doi: 10.1038/s41565-023-01336-z (PMC10359187; doi:10.1038/s41565-023-01336-z)
Supplement: Supplementary file 1 — Supplementary Sections 1–3, Fig. 1 and refs. 1–14. [file 41565_2023_1336_MOESM1_ESM.pdf]

nature nanotechnology

Article https://doi.org/10.1038/s41565-023-01336-z

Chiral antiferromagnetic Josephson junctions as spin-triplet supercurrent spin valves and d.c. SQUIDs

In the format provided by the authors and unedited

Supplementary Information

Chiral antiferromagnetic Josephson junctions as spin-triplet supercurrent spin-valves and dc SQUIDs

Kun-Rok Jeon[1,2*], Binoy Krishna Hazra[1], Jae-Keun Kim[1], Jae-Chun Jeon[1], Hyeon Han[1], Holger L. Meyerheim[1], Takis Kontos[3], Audrey Cottet[3*] and Stuart S. P. Parkin[1*]

[1]*Max Planck Institute of Microstructure Physics, Weinberg 2, 06120 Halle (Saale), Germany*

[2]*Department of Physics, Chung-Ang University (CAU), Seoul, Republic of Korea*

[3]*Laboratoire de Physique de l'Ecole normale supérieure, ENS, Université PSL, CNRS, Sorbonne Université, Université de Paris, F-75005 Paris, France*

*To whom correspondence should be addressed: jeonkunrok@gmail.com, Audrey.Cottet@phys.ens.fr, stuart.parkin@halle-mpi.mpg.de

This PDF file includes:

Supplementary Text

Fig. S1

References (*S1-S14*)

Section S1: Quasiclassical theory of superconducting proximity effect and spin-triplet supercurrent SV effect.

As outlined in Methods, we develop in this section the quasiclassical theory of the superconducting proximity effect in a conventional AFM and in a chiral AFM. We derive the equations describing the propagation of superconducting correlations in the diffusive limit – Usadel equations – which are relevant to the devices studied experimentally here. We find that *all the superconducting correlations* of spin-unpolarised singlets ($S = 0$), spin-zero ($S = 1$, $m_s = 0$) and spin-polarised triplets ($S = 1$, $m_s = \pm 1$) are strongly damped by exchange spin-splitting fields in the conventional AFM, leading to a short-ranged proximity effect. In case of the chiral AFM, the spin-momentum locking along with the Weyl node structure turns out to cause a qualitatively different superconducting proximity effect. The spin-momentum locking implies that the spin-texturing in the chiral AFM plays the role of a vector potential, thereby phase-shifting the superconducting order parameter and inducing a φ-junction (or π-junction) behaviour, which is controlled by modulating the chiral antiferromagnetic-spin texturing. We also note that the Weyl node structure (directly relevant to the Berry curvature) imposes the existence of spin triplet correlations inside the chiral AFM [see Eq. (S16)]. These correlations are expected to propagate over a long distance [see Eq. (S17)]. Notably, when the OOP magnetic field is applied and swept, the amplitude I_{Chiral} and phase $\varphi_{0,chiral}$ of Josephson triplet supercurrent through the d_s > 150 nm Mn_3Ge barrier can both visibly change because *these values scale directly with d_s and are determined by how the antiferromagnetic-spin texture of Mn_3Ge is configured* [Eq. (S28)]. This is the theoretical insight that explains qualitatively all the experimental findings of the present paper. Full details of our quasiclassical theory, which reproduces the hysteretic Fraunhofer pattern (Fig. 1d) and explains the 0-to-π phase shift in the SQUID data (Extended Data Fig. 3a,b), will be presented below.

We consider two different AFMs of topological origin: the conventional itinerant AFM such as IrMn, and the chiral AFM corresponding to our Mn_3Ge. The two Hamiltonians are markedly different. Following Krivoruchko[S1], the minimal Hamiltonian in the conventional AFM reads:

$$\check{H}_{AFM} = \int d^3r \sum_{\sigma} \left(\psi_\sigma^\dagger(\vec{r}) \left[-\frac{\hbar^2\nabla^2}{2m} - \mu \right] \psi_\sigma(\vec{r}) + \varphi_\sigma^\dagger(\vec{r}) \left[\frac{\hbar^2\nabla^2}{2m} + \mu \right] \varphi_\sigma(\vec{r}) \right)$$

$$+\int d^3r\, E_{exc} \sum_\sigma \left(\psi_\sigma^\dagger(\vec{r}) \varphi_{-\sigma}(\vec{r}) + \psi_{-\sigma}(\vec{r}) \varphi_\sigma^\dagger(\vec{r}) \right) \qquad \text{(S1)}$$

where E_{exc} is the exchange field characterizing the magnetization of the AFM and $\psi_\sigma^\dagger(r)$, $\Psi_\sigma(\vec{r})$, $\varphi_\sigma^\dagger(\vec{r})$ and $\varphi_\sigma(\vec{r})$ are the field operators for electrons with spin $\sigma \in \{\uparrow, \downarrow\}$ at position $\vec{r}$ in the two sublattices of the AFM. In the case of a chiral AFM, the Weyl nodes give rise to a two-orbital structure and a minimal Hamiltonian can be written as[S2,S3]:

$$\widehat{H}_{ChAFM} = \int d^3r \left(\psi_\uparrow^\dagger(\vec{r}), \psi_\downarrow^\dagger(\vec{r}), \varphi_\uparrow^\dagger(\vec{r}), \varphi_\downarrow^\dagger(\vec{r}) \right) \left[-i\hbar v_F \check{\tau}_3 \vec{\nabla}.\vec{\sigma} - J\vec{\mathrm{M}}(r).\vec{\sigma} \right] \begin{pmatrix} \psi_\uparrow(\vec{r}) \\ \psi_\downarrow(\vec{r}) \\ \varphi_\uparrow(\vec{r}) \\ \varphi_\downarrow(\vec{r}) \end{pmatrix} \qquad \text{(S2)}$$

where $\check{\tau}_{1,2,3}$ are Pauli matrices acting in the chirality (orbital) space of the AFM, $\vec{\sigma} = (\check{\sigma}_1, \check{\sigma}_2, \check{\sigma}_3)$ is a spatial vector which encloses the spin Pauli matrices, v_F is the Fermi velocity, J is the exchange interaction and $\overrightarrow{\delta\mathrm{M}}(\vec{r})$ is the magnetic texture of the AFM. From Eq.(S2), if the uniform component M_0 of the magnetization is along the *z*-axis, the Weyl nodes will be located along the *z*-axis of the spin states (corresponding to $\check{\sigma}_3$) at $p_z = \pm J M_0 / v_F$. We will see below that the spin-momentum locking, the Weyl nodes and the associated magnetic texture deeply modify the superconducting proximity effect with respect to the conventional AFM case.

We now study the physical consequences of the two above Hamiltonians. More precisely, our aim is to study the spatial propagation of superconducting correlations in the presence of the antiferromagnetic order, in the diffusive regime which is relevant for our experiment. To describe the superconducting proximity effect, one can use the matrix Gorkov Green's function

$\hat{G}$ defined as:

$$\hat{G}(\vec{r}_1, \vec{r}_2, t_1, t_2) = -i\theta(t_1 - t_2)\langle\{\Psi(\vec{r}_1, t_1), \Psi^\dagger(\vec{r}_2, t_2)\}\rangle \qquad \text{(S3)}$$

where $\langle\ \rangle$ denotes the thermal average, $\{\ \}$ is the anticommutator, and $\Psi(\vec{r}, t) = \left(\psi_\uparrow(\vec{r}, t), \psi_{\uparrow\downarrow}(\vec{r}, t), \psi_\uparrow^\dagger(\vec{r}, t), \psi_\downarrow^\dagger(\vec{r}, t), \varphi_\uparrow(\vec{r}, t), \varphi_\downarrow(\vec{r}, t), \varphi_\uparrow^\dagger(\vec{r}, t), \varphi_\downarrow^\dagger(\vec{r}, t)\right)$. Due to the appearance of the field operators and their conjugates in $\Psi(\vec{r})$, the Green's function has a structure not only in the spin and orbital (or chiral) space but also in the Nambu (electron/hole) space, and the matrix Green's function contains both the normal and 'anomalous' (superconducting) correlations. For later use we define spin and orbital Pauli matrices extended to the spin⊗orbital⊗Nambu space, defined as $\hat{\sigma}_3 = diag[1, -1, 1, -1, 1, -1, 1, -1]$ and $\hat{\tau}_3 = diag[1, 1, 1, 1, -1, -1, -1, -1]$ respectively, and Nambu Pauli matrices such as $\hat{\rho}_3 = diag[1, 1, -1, -1, 1, 1, -1, -1]$ (the other Pauli matrices can be defined accordingly). For simplicity we use below $\hbar$=1. Note that we consider only the retarded Green's function because we study supercurrents which are equilibrium quantities. Since we consider a stationary problem we make the Fourier time transform $\hat{G}(\vec{r}_1, \vec{r}_2, \varepsilon) = \int dt e^{i\varepsilon(t_1 - t_2)} \hat{G}(\vec{r}_1, \vec{r}_2, t_1, t_2)$.

We start with the case of the AFM. It is important to specify that even though we consider a uniform AFM, the interface between superconductor (SC) and AFM can have a finite (and non-colinear) magnetization, depending on their microscopic details. Such an interface (uncompensated) magnetization could induce the spin-rotation process which in turn leads to the conversion of the singlet superconducting correlations into triplet correlations with either spin 0 or spin 1[S7]. It is thus essential to study the propagation of all types of the superconducting correlations in the AFM. This description can be carried out by generalizing the quasiclassical theory of superconductivity. For this purpose, it is convenient to redefine the Gorkov Green's function as $\acute{G}(\vec{r}_1, \vec{r}_2, t_1, t_2) = \hat{\rho}_3 \hat{\tau}_3 \hat{G}(\vec{r}_1, \vec{r}_2, t_1, t_2)$. This Green's function $\acute{G}$ follows the Gorkov equations:

$$\left(\varepsilon\hat{\rho}_3\hat{\tau}_3 + \frac{\hbar^2\nabla^2_{\vec{r}_1}}{2m} + \mu - E_{exc}\hat{\tau}_1\hat{\tau}_3\hat{\sigma}_1 + \hat{S}_{imp}\right)\check{G}(\vec{r}_1,\vec{r}_2,\varepsilon) = \hat{1}\delta(\vec{r}_1,\vec{r}_2) \qquad \text{(S4)}$$

$$\check{G}(\vec{r}_1,\vec{r}_2,\varepsilon)\left(\varepsilon\hat{\rho}_3\hat{\tau}_3 + \frac{\hbar^2\nabla^2_{\vec{r}_2}}{2m} + \mu - E_{exc}\hat{\tau}_1\hat{\tau}_3\hat{\sigma}_1 + \hat{S}_{imp}\right) = \hat{1}\delta(\vec{r}_1,\vec{r}_2) \qquad \text{(S5)}$$

where $\hat{1}$ is the identity in the spin⊗orbital⊗Nambu space. These equations can be obtained by writing down the equations of motion for the field operators due to the Hamiltonian $\hat{H}_{AFM}$. We have added a self-energy term $\hat{S}_{imp}$ which accounts for electronic scattering on the impurities of the material, along the standard approach of the quasiclassical theory of superconductivity. In the limit where the electronic correlations evolve on a characteristic scale much larger than the Fermi wavelength, one can simplify the Gorkov description by making a quasiclassical approximation. We define the quasiclassical Eilenberger Green's function in the mixed representation:

$$\hat{g}_\varepsilon(\vec{R},\vec{n}) = \frac{i}{\pi}\oint d\xi_p \int \frac{d^3\vec{r}}{(2\pi)^3} e^{-i\vec{p}.\vec{r}}\check{G}(\vec{R}+\vec{r}/2\,;\vec{R}-\vec{r}/2\,,\varepsilon) \qquad \text{(S6)}$$

Due to the integration with respect to $\xi_p = p^2/2m$, $\hat{g}_\varepsilon$ depends only on the direction $\vec{n}$=$\vec{p}/p$ of $\vec{p}$. Making use of the gradient expansion technique and performing a semiclassical approximation, one obtains the following Eilenberger equation for the AFM case [S4-S6]:

$$-iv_F\vec{n}.\vec{\nabla}_R\hat{g}_\varepsilon(\vec{R},\vec{n}) = \left[\varepsilon\hat{\rho}_3\hat{\tau}_3 - \hat{\tau}_1\hat{\tau}_3\hat{\sigma}_1E_{exc} + \hat{S}_{imp}, \hat{g}_\varepsilon(\vec{R},\vec{n})\right] \qquad \text{(S7)}$$

Equation (S7) is the same as the one found by Krivoruchko[S1] but with triplet correlations explicitly taken into account. We now define the isotropic Green's function:

$$\hat{\mathcal{G}}_\varepsilon(\vec{R}) = \int\frac{d\Omega}{4\pi}\hat{g}_\varepsilon(\vec{R},\vec{n}) \qquad \text{(S8)}$$

where $\int\frac{d\Omega}{4\pi}$ is an angular integration on the direction $\vec{n}$. This last Green's function is a relevant quantity when impurity scattering in the material is strong. The impurities yield an isotropization of the electronic correlations[S4], which leads to the Usadel equation:

$$D\vec{\nabla}_R.\hat{\mathcal{G}}_\varepsilon(\vec{R})\vec{\nabla}_R\hat{\mathcal{G}}_\varepsilon(\vec{R}) = \left[-i\varepsilon\hat{\rho}_3\hat{\tau}_3 + i\hat{\tau}_1\hat{\tau}_3\hat{\sigma}_1E_{exc}, \hat{\mathcal{G}}_\varepsilon(\vec{R})\right] \qquad \text{(S9)}$$

where D is the diffusion constant in the material. Equation (S9) resembles that for ferromagnetic metals but with the major difference that the exchange field term in E_{exc} is off-

diagonal in orbital and in spin, which leads to substantial differences in the proximity effect.

It is instructive to start by considering the normal state bulk solution of Eq. (S9). In fact, the simplest method is to calculate the bulk value of $\acute{G} = \acute{G}_0$ given by Eqs. (S4) and (S5) without $\hat{S}_{imp}$, because in the homogeneous diffusive case $\hat{\mathcal{G}}_\varepsilon = \hat{g}_\varepsilon = \frac{i}{\pi}\oint d\xi_p\, \acute{G}_0$ is expected. We have checked that the obtained $\hat{\mathcal{G}}_\varepsilon$ is consistent with Eq.(S9) and with the normalization condition ${\hat{\mathcal{G}}_\varepsilon}^2$=$\hat{1}$. One has matrix elements:

$$\mathcal{G}_{\psi_\sigma^\dagger\psi_\sigma} = \mathcal{G}_{\varphi_\sigma\varphi_\sigma^\dagger} = \frac{i\varepsilon}{\sqrt{E_{exc}^2-\varepsilon^2}} \qquad \text{(S10a)}$$

$$\mathcal{G}_{\psi_\sigma\psi_\sigma^\dagger} = \mathcal{G}_{\varphi_\sigma^\dagger\varphi_\sigma} = \frac{-i\varepsilon}{\sqrt{E_{exc}^2-\varepsilon^2}} \qquad \text{(S10b)}$$

$$\mathcal{G}_{\varphi_{\bar{\sigma}}\psi_\sigma^\dagger} = \mathcal{G}_{\varphi_\sigma^\dagger\psi_{\bar{\sigma}}} = \frac{iE_{exc}}{\sqrt{E_{exc}^2-\varepsilon^2}} \qquad \text{(S10c)}$$

$$\mathcal{G}_{\psi_{\bar{\sigma}}\varphi_\sigma^\dagger} = \mathcal{G}_{\psi_{\bar{\sigma}}^\dagger\varphi_\sigma} = \frac{-iE_{exc}}{\sqrt{E_{exc}^2-\varepsilon^2}} \qquad \text{(S10d)}$$

No superconducting correlations are present in $\hat{\mathcal{G}}_\varepsilon$ at this stage since it can only occur due to proximity effect, hence terms like $\mathcal{G}_{\psi_\sigma^\dagger\psi_\sigma^\dagger}$ are zero.

We now consider the spatial evolution of the electronic correlations in the case where the conventional AFM is contacted to the SC. In order to get qualitative insights, it is useful to consider the experimentally relevant limiting case of a weak superconductivity proximity effect[S4]. Using a 1D geometry to simplify the discussion, the equations governing the spatial evolution of the triplet and singlet proximity effect, which arise from Eq. (S9), can be approximated as:

$$D\mathcal{G}_{\psi_\sigma^\dagger\psi_\sigma}\partial_x^2\mathcal{F}_{\psi_\sigma^\dagger\psi_\sigma^\dagger} = 2i\varepsilon\mathcal{F}_{\psi_\sigma^\dagger\psi_\sigma^\dagger} \qquad \text{(S11a)}$$

$$D\mathcal{G}_{\psi_\sigma^\dagger\psi_\sigma}\partial_x^2\mathcal{F}_{\psi_\sigma^\dagger\psi_{\bar{\sigma}}^\dagger} = 2i\varepsilon\mathcal{F}_{\psi_\sigma^\dagger\psi_{\bar{\sigma}}^\dagger} \qquad \text{(S11b)}$$

$$D\mathcal{G}_{\psi_\sigma\psi_\sigma^\dagger}\partial_x^2\mathcal{F}_{\psi_\sigma\psi_\sigma} = -2i\varepsilon\mathcal{F}_{\psi_\sigma\psi_\sigma} \qquad \text{(S11c)}$$

$$D\mathcal{G}_{\psi_\sigma\psi_\sigma^\dagger}\partial_x^2\mathcal{F}_{\psi_\sigma\psi_{\bar{\sigma}}} = -2i\varepsilon\mathcal{F}_{\psi_\sigma\psi_{\bar{\sigma}}} \qquad \text{(S11d)}$$

Similar equations arise for the φ_σ states, with the energy ε replaced by -ε. Above, for clarity, we note $\mathcal{G}$ the matrix elements of $\hat{\mathcal{G}}_\varepsilon$ which describe ordinary correlations and $\mathcal{F}$ the elements which involve superconducting correlations (for instance, $\mathcal{F}_{\psi_\sigma^\dagger\psi_\sigma^\dagger} = \mathcal{G}_{\psi_\sigma^\dagger\psi_\sigma^\dagger}$). The above equations show that, in accordance to intuition, the antiferromagnetic order is averaged out from the point of view of the Cooper pairs [E_{exc} does not appear in Eq. (S11)]. This phenomenon is essentially different from what happens in a ferromagnet (FM). Indeed, in the FM, E_{exc} could appear in the right members of Eq. (S11) in such a way that it limits the propagation of spin-unpolarised singlet ($S=0$, $m_s = 0$) and spin-zero triplet ($S=1$, $m_s = 0$) correlations whereas the scale ε only controls the propagation of spin-polarised triplet correlations. Specifically, the exchange field combined with disorder produces a dephasing of spins up with respect to spin down and this is why superconducting correlations involving opposite spins are damped by a strong exchange field, but this is not the case for the spin-polarised triplets ($S=1$, $m_s=\pm 1$). In the AFM case, the propagation of superconducting correlations is distinctively different since the right member of Eq. (S11) involves only the scale ε for all types of correlations. One could expect that all correlations are long-ranged for this reason. However, the superconducting proximity effect in the AFM is indirectly damped because the antiferromagnetic exchange field strongly affects the value of normal electronic correlations. Indeed, there is a strong reduction factor provided by the $\mathcal{G}$ terms in all the left members of Eq. (S11). These factors scale with ε/E_{exc} for a large E_{exc}. To shed light on this effect, we now make the experimentally relevant assumption that the exchange field is strong ($|E_{exc}| \gg \varepsilon$), in order to simplify the equations. Using the weak proximity effect means that in Eq.(S11), we can replace the normal correlation terms such as $\mathcal{G}_{\psi_\sigma^\dagger\psi_\sigma}$ by their bulk value given in (S10). This gives:

$$D\partial_x^2\mathcal{F}_{\psi_\sigma^\dagger\psi_\sigma^\dagger} = 2|E_{exc}|\mathcal{F}_{\psi_\sigma^\dagger\psi_\sigma^\dagger} \qquad \text{(S12a)}$$

$$D\partial_x^2\mathcal{F}_{\psi_\sigma^\dagger\psi_{\bar{\sigma}}^\dagger} = 2|E_{exc}|\mathcal{F}_{\psi_\sigma^\dagger\psi_{\bar{\sigma}}^\dagger} \qquad \text{(S12b)}$$

$$D\partial_x^2\mathcal{F}_{\varphi_\sigma\,\varphi_\sigma} = 2|E_{exc}|\mathcal{F}_{\varphi_\sigma\,\varphi_\sigma} \qquad \text{(S12c)}$$

$$D\partial_x^2\mathcal{F}_{\varphi_\sigma\,\varphi_{\bar{\sigma}}} = 2|E_{exc}|\mathcal{F}_{\varphi_\sigma\,\varphi_{\bar{\sigma}}} \qquad \text{(S12d)}$$

and identical equations for the φ_σ and ψ_σ sectors. These equations show that the superconducting correlations are exponentially damped on the characteristic length $\xi_{AFM} = \sqrt{\frac{\hbar D}{2|E_{exc}|}}$. Contrary to the FM case, all the superconducting correlations of spin-unpolarised singlets ($S = 0$), spin-zero ($S = 1$, $m_s = 0$) and spin-polarised triplets ($S = 1$, $m_s = \pm 1$) in the AFM are affected in the same way and decay over a quite short length scale without an oscillatory behaviour. This generalizes the findings of Krivoruchko to the case where triplet proximity effect is present in the system. Even if one could assume that there exists a spin-active interface which causes the appearance of the spin-polarised triplets ($S = 1$, $m_s = \pm 1$) in the AFM to a certain extent, the propagation of these correlations is in the end expected to be short-ranged.

We now turn to the case relevant for Mn_3Ge where the AFM is described by a Weyl semimetal model (chiral AFM). The band structure of Mn_3Ge is a priori complex with several pairs of Weyl nodes. Nevertheless, it is enough for our purpose to model it with a minimal model of two Weyl nodes in a Weyl semimetal with a magnetic texture. The Weyl node structure and the spin-momentum locking have profound consequences. The quasiclassical procedure has to be modified due to the different symmetries of the system Hamiltonian. We now use

$$\hat{g}_\varepsilon(\vec{R},\vec{n}) = \frac{i}{\pi}\hat{\rho}_3\hat{\tau}_3 U \oint d\xi_p \int \frac{d^3\vec{r}}{(2\pi)^3} e^{-i\vec{p}.\vec{r}}\hat{G}(\vec{R}+\vec{r}/2\,;\vec{R}-\vec{r}/2\,,\varepsilon)U \qquad \text{(S13)}$$

with

$$U = \hat{\sigma}_0\left(\frac{\hat{\rho}_0+\hat{\rho}_3}{2}\right) + \hat{\sigma}_2\left(\frac{\hat{\rho}_0-\hat{\rho}_3}{2}\right) \qquad \text{(S14)}$$

This gives the Eilenberger equation

$$\{\tfrac{v_F}{2}\vec{\sigma}.\vec{\nabla}_R, \hat{g}_\varepsilon(\vec{R},\vec{n})\} = \big[i\varepsilon\hat{\rho}_3\hat{\tau}_3 - iv_F p_F \vec{n}.\vec{\sigma} + iJM_0\hat{\rho}_3\hat{\tau}_3\hat{\sigma}_3 + iJ\hat{\rho}_3\hat{\tau}_3\overrightarrow{\delta\mathrm{M}}(\vec{R}).\vec{\sigma} + $$

$$+\hat{S}'_{imp}, \hat{g}_\varepsilon(\vec{R},\vec{n})\big] \qquad \text{(S15)}$$

where the Pauli matrix $\hat{\tau}_3$ now acts in the chirality subspace and we have added the self energy $\hat{S}'_{imp}$ of scattering impurites. The above equation is similar to that obtained recently for surface states of topological insulators except for the Weyl node structure[S5,S6]. The spin-chirality structure as well as its normalization may be found like in these works by looking at the dominant term in the commutator of the right-hand side of Eq. (S15). For well-defined Weyl cones, $JM_0\hat{\rho}_3\hat{\tau}_3\hat{\sigma}_3$ is a large term[S3] which means that the superconducting correlations acquire a spin-chirality structure in the chiral AFM [S3]. Similarly to the case of topological insulators [S5,S6], we project the quasiclassical Green's function using the angle –dependent projector $\frac{1+\vec{n}.\vec{\sigma}}{2}$. Such a spin structure implies that there are triplet superconducting correlations. In order to derive the Usadel equations, we now expand in spherical harmonics the Eilenberger Green's function using the expression:

$$\hat{g}_\varepsilon(\vec{R},\vec{n}) = \{\hat{\mathcal{G}}_\varepsilon(\vec{R}) + \vec{n}.\vec{h}_\varepsilon(\vec{R})\}\tfrac{1+\vec{n}.\vec{\sigma}}{2} \qquad \text{(S16)}$$

where $\hat{\mathcal{G}}_\varepsilon(\vec{R})$ and $\vec{h}_\varepsilon(\vec{R})$ have no structure in the spin space (here we mean the new spin space which goes along with the Green's function transformation of Eqs. (S13) and (S14)). We also assume that $\hat{\mathcal{G}}_\varepsilon(\vec{R})$ commutes with $\hat{\tau}_3$. The normalization of $\hat{g}_\varepsilon(\vec{R},\vec{n})$ requires $\hat{\mathcal{G}}_\varepsilon(\vec{R})^2 = 1$ and $\{\hat{\mathcal{G}}_\varepsilon(\vec{R}), \vec{h}_\varepsilon(\vec{R})\} = 1$. Injecting expression (S16) in Eq. (S15), using the usual derivation method of Usadel equation and taking a spin trace, we get finally:

$$D\vec{\tilde{\nabla}}_R.\hat{\mathcal{G}}_\varepsilon(\vec{R})\vec{\tilde{\nabla}}_R\hat{\mathcal{G}}_\varepsilon(\vec{R}) = [-i\varepsilon\hat{\rho}_3\hat{\tau}_3, \hat{\mathcal{G}}_\varepsilon(\vec{R})] \qquad \text{(S17)}$$

with

$$\vec{\tilde{\nabla}}_R(x) = \vec{\nabla}_R(x) - i\frac{J\vec{\mathrm{M}}(\vec{R})}{v_F}[\hat{\rho}_3\hat{\tau}_3, x] \qquad \text{(S18a)}$$

$$\vec{\tilde{\nabla}}_R.\vec{x} = \vec{\nabla}_R.\vec{x} - i\frac{J\vec{M}(\vec{R})}{v_F}.[\hat{\rho}_3\hat{\tau}_3, \vec{x}] \qquad \text{(S18b)}$$

and $D = \frac{\tau v_F^2}{3}$ the diffusion constant in the material and $\vec{M}(\vec{R}) = \overrightarrow{M_0} + \overrightarrow{\delta M}(\vec{R})$. Equation (S17) is very similar to the form found for topological insulator surface states. The right hand side does not involve any characteristic energy as a consequence of spin-momentum locking. This implies that the magnetic texture plays the role of a vector potential which appears in (S18). The expression of the average current flowing through the junction can be expressed as:

$$I = \frac{-i\pi e}{8} k_B T D \sum_{\omega_n} Tr\left[\hat{\rho}_3 \hat{\mathcal{G}}_{\omega_n}(\vec{R}) \vec{\tilde{\nabla}}_R \hat{\mathcal{G}}_{\omega_n}(\vec{R})\right] \qquad \text{(S19)}$$

Above, we use Usadel Green's functions which depend on the Matsubara frequencies $\omega_n = (2n+1)\pi k_B T$ instead of the real energy ε because it will simplify the calculation of the current (one can formally relate $\hat{\mathcal{G}}_\omega$ and $\hat{\mathcal{G}}_\varepsilon$ using $-i\varepsilon \to \omega$). The value of I depends both on the position of the Weyl nodes (located at $\vec{k} = (0,0,\pm\frac{JM_0}{\hbar v_F})$) and on the effective axial magnetic field $\vec{B}_{axial} = \vec{\nabla}_R \times \overrightarrow{\delta M}(\vec{R})$. It is instructive to solve the above problem for $\overrightarrow{\delta M}(\vec{R}) = 0$, to put forward the effect of the Weyl nodes, which are directly related to the Berry curvature, and to get a simple idea of the functional form of the supercurrent in Mn_3Ge. We work in the weak proximity effect (like in the previous case) for each chirality sector. We thus take as before the bulk value for the normal part of $\hat{\mathcal{G}}_\omega$, and we note:

$$\hat{\mathcal{G}}_\omega(\vec{R}) = \begin{bmatrix} sgn\,(\omega_n) & \mathcal{F}_+(\vec{R}) \\ \mathcal{F}_-(\vec{R}) & -sgn\,(\omega_n) \end{bmatrix} \otimes \hat{\tau}_3 \otimes \hat{\sigma}_3$$

We get the following equations for the anomalous parts of the Green's functions:

$$\hbar D \left(\partial_z \mp 2i\frac{JM_0}{\hbar v_F}\tau\right)^2 \mathcal{F}_\pm = 2\omega_n\, sgn\,(\omega_n)\mathcal{F}_\pm \qquad \text{(S20a)}$$

where τ is the chirality quantum number. We have assumed here that $\vec{M}(\vec{R}) = M_0\hat{z}$ and $\mathcal{F}_\pm(\vec{R}) = \mathcal{F}_\pm(z)$. We also use the following boundary conditions:

$$\left(\partial_z \mp 2i\frac{JM_0}{\hbar v_F}\tau\right)\mathcal{F}_\pm(z = -\frac{d}{2}) = (\gamma + \chi\tau)\frac{\Delta e^{\mp i\varphi/2}}{\sqrt{\Delta^2 - \omega_n{}^2}} \qquad \text{(S20b)}$$

$$\left(\partial_z \mp 2i\frac{JM_0}{\hbar v_F}\tau\right)\mathcal{F}_\pm\left(z=+\frac{d}{2}\right) = -(\gamma+\chi\tau)\frac{\Delta e^{\pm i\varphi/2}}{\sqrt{\Delta^2-\omega_n{}^2}} \qquad \text{(S20c)}$$

which relate $\mathcal{F}_\pm(z)$ to the value of the superconducting correlations in the superconductor. These boundary conditions are adapted from those of Ref. S9. The parameter γ accounts for the transparency of the tunnel barrier and the parameter χ accounts for the chirality dependence of the barrier. The chirality dependence of the boundary conditions for quasiclassical Green's functions is natural. It is reminiscent of chirality blockade predicted by Bovenzi et al.[S3] Deriving the full spin/chiral dependent boundary conditions is beyond the scope of this section[S8] and will be the subject of a subsequent theoretical work[S10]. Nevertheless, given the structure of the problem which is diagonal in the chiral index, it is reasonable, in the regime we consider, to use the above ansatz that the barrier has one chirality independent term and one term proportional to $\hat{\tau}_3$. The solutions of the differential equations (S20a), (S20b) and (S20c) are functions of the type:

$$\mathcal{F}_s = \mathcal{A}_s\, e^{z\sqrt{2|\omega_n|/\hbar D}}e^{is\tau 2JM_0/\hbar v_F} + \mathcal{B}_s\, e^{-z\sqrt{2|\omega_n|/\hbar D}}e^{is\tau 2JM_0/\hbar v_F} \qquad \text{(S21)}$$

with $s=\pm1$. Note that $\mathcal{F}_+(z)$ and $\mathcal{F}_-(z)$ have both spatially increasing and decreasing components, as expected for a finite size layer. Letting d be the length of the junction along the Weyl node vectors and defining $k_n=\sqrt{2|\omega_n|/\hbar D}$, we get the following expression for the current:

$$I = k_B T\sum_{\omega_n,\tau}(\gamma+\chi\tau)^2\frac{\Delta^2}{\omega_n{}^2+\Delta^2}k_n{}^{-1}\,\mathrm{csch}\,(k_n d)\sin\left(\varphi+d\frac{2JM_0}{\hbar v_F}\tau\right) \qquad \text{(S22)}$$

where Δ is the superconducting gap in the superconducting electrodes. One can draw important conclusions at this stage and also test experimentally formula (S22) using the Fraunhoffer pattern. In the case where $\chi=0$, we can further simplify the above formula to:

$$I = I_C\sin(\varphi)\cos\left(d\frac{2JM_0}{\hbar v_F}\right) \qquad \text{(S23)}$$

with $I_C = k_B T\sum_{\omega_n,\tau}(\gamma+\chi\tau)^2\frac{\Delta^2}{\omega_n{}^2+\Delta^2}k_n{}^{-1}\,\mathrm{csch}\,(k_n d)$. For $d\frac{2JM_0}{\hbar v_F}>\pi/2$, the Josephson junction becomes a π junction. Interestingly, a phase shift close to π is experimentally observed

in our data (Fig. 4 of the main text and Extended Data Fig. 3).

When there is a magnetic texture in the system and when the barrier parameter χ is non-zero, like in the Mn_3Ge, the peculiar band structure of the Weyl semimetal has another important consequence. Using the exact band structure of Mn_3Ge is beyond the scope of the present study but the use of a simplified magnetic texture in addition to the constant Weyl points is very insightful regarding both the understanding of the Fraunhoffer patterns and the damping of the supercurrent as a function of superconducting contact separation d. We assume specifically that, in addition to the constant magnetization along the *z*-axis, there are two spatially-dependent components forming the spin texture along the *x*- and *z*-axis. For the sake of simplicity, we take a domain wall induced spin texture:

$$\vec{\mathrm{M}}(\vec{R}) = [\delta M \,\mathrm{sech}(Qy), 0, M_0 + \delta M \tanh(Qy)] \qquad \text{(S24)}$$

where Q is the inverse antiferromagnetic domain size and δM is the amplitude of the magnetization of the texture. In such conditions, Eq. (S20a) is modified as:

$$\hbar D \left(\partial_z \mp i\frac{JM_0}{\hbar v_F}\tau \mp i\frac{J\delta M \tanh(Qy)}{\hbar v_F}\tau\right)^2 \mathcal{F}_\pm - \left(\frac{J\delta M\,\mathrm{sech}(Qy)}{\hbar v_F}\right)^2 \mathcal{F}_\pm = 2\omega_n\, sgn\,(\omega_n)\mathcal{F}_\pm \qquad \text{(S25)}$$

The existence of the magnetic texture has two important consequences regarding the supercurrent expression in the general case. The longitudinal component of the texture provides spatially dependent and chirality dependent phase shifts in Eq. (S21) whereas the transverse part provides an exponential damping of the superconducting proximity effect. Contrarily to the ferromagnetic case, only the transverse part of the magnetic texture can damp the superconductivity as a consequence of spin momentum locking [S5,S6]. The general form of the supercurrent arising from Eq. (S25) when a magnetic field is applied to the junction is quite cumbersome. Nevertheless, in the large domain limit which is experimentally realistic in our case[S7], i.e. $Qw \ll 1$ with w the junction width along the *y*-axis, one can get insightful expressions [S5,S6]:

$$I \approx \int_{-\frac{w}{2}}^{\frac{w}{2}} \frac{dy}{w} k_B T \sum_{\omega_n,\tau} (\gamma + \chi\tau)^2 \frac{\Delta^2}{\omega_n{}^2+\Delta^2} k_n{}^{-1} \operatorname{csch}(k_n d) \sin\left(\varphi + d\frac{2JM_0}{\hbar v_F}\tau + d\frac{2J\delta M}{\hbar v_F} Qy\tau + 2\pi\frac{\Phi}{\Phi_0}\frac{y}{w}\right) \qquad \text{(S26)}$$

with $k_n = \sqrt{\frac{2|\omega_n|}{\hbar D} + \left(\frac{2J\delta M}{\hbar v_F}\right)^2}$.

This allows us to write how the magnetic texture modifies Eq. (S22):

$$I \approx k_B T \sum_{\omega_n,\tau} (\gamma + \chi\tau)^2 \frac{\Delta^2}{\omega_n{}^2+\Delta^2} k_n{}^{-1} \operatorname{csch}(k_n d) \sin\left(\varphi + d\frac{2JM_0}{\hbar v_F}\tau\right) \operatorname{sinc}\left(\pi\frac{\Phi}{\Phi_0} + Qwd\frac{J\delta M}{\hbar v_F}\tau\right) \qquad \text{(S28)}$$

This yields the following expression for the critical current, found as the maximum of I as a function of φ:

$$I_c = I_0\left(\left(\sum_\tau (1 + \tau\frac{2\chi\gamma}{\gamma^2+\chi^2}) \sin\left(d\frac{2JM_0}{\hbar v_F}\tau\right) \operatorname{sinc}\left(\pi\frac{\Phi}{\Phi_0} + Qwd\frac{J\delta M}{\hbar v_F}\tau\right)\right)^2 + \left(\sum_\tau (1 + \tau\frac{2\chi\gamma}{\gamma^2+\chi^2}) \cos\left(d\frac{2JM_0}{\hbar v_F}\tau\right) \operatorname{sinc}\left(\pi\frac{\Phi}{\Phi_0} + Qwd\frac{J\delta M}{\hbar v_F}\tau\right)\right)^2\right)^{1/2} \qquad \text{(S29)}$$

Equation (S29) shows that the antiferromagnetic-spin texture shifts the Fraunhofer pattern by a chirality dependent phase $Qwd\frac{J\delta M}{\hbar v_F}\tau$. Since this phase term is proportional to $Q\delta M$, the current I is a hysteretic function of the applied magnetic field with the characteristic mirror symmetric behavior with respect to zero magnetic field. The resulting Fraunhofer patterns are shown in Fig. 1d (on the top of the experimental data, main text) and Fig. S1 as well. They account well for the peculiar hysteretic shifts in Fig. 1d of the main text. The hysteresis of the Fraunhofer pattern stems both from the fact that there is a chirality dependent phase shift arising from the antiferromagnetic-spin texture and that the interface parameter χ is non-zero. Important aspects of Eq. (S22) and its generalization in the textured case under a finite OOP magnetic field [Eq. (S28)] are therefore experimentally tested in Fig. 1d (of the main text).

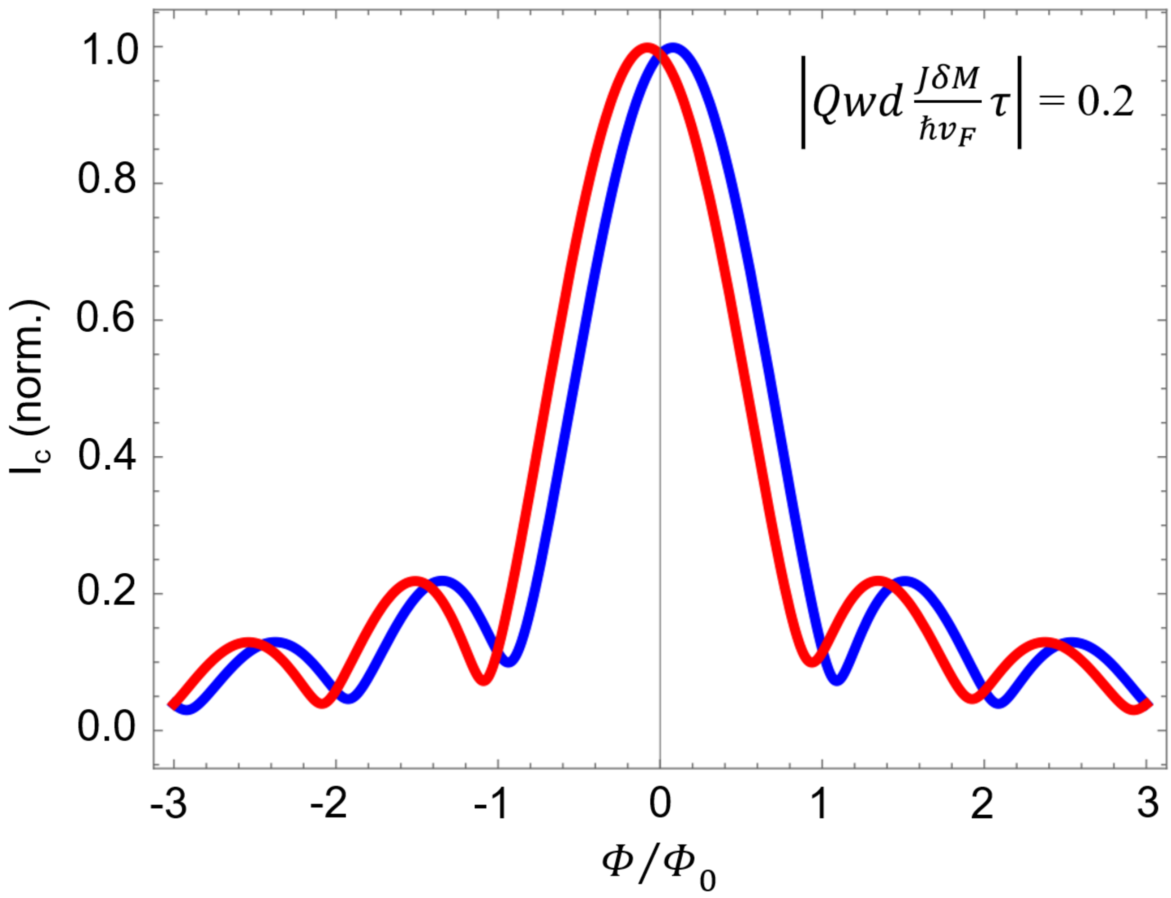

Figure S1. Calculated hysteretic Fraunhofer pattern using Eq. (S29) with $\left|Qwd\frac{J\delta M}{\hbar v_F}\tau\right| = 0.2$, which basically reproduces Fig. 1d of the main text.

In addition, from the above theory, it follows that, in general (as in the topological insulator case [S5,S6]), the CPR of a chiral antiferromagnet Josephson junction has both the critical current $I_{C,chiral}$ and a characteristic phase $\varphi_{0,chiral}$ which depend on the position of the Weyl nodes and on the magnetic texture configuration (through M_0 in particular) which can be changed when the external magnetic field is swept. In our case, the specific expressions of I_{Chiral} and $\varphi_{0,chiral}$ depend on microscopic details of the interface and the bulk of Mn_3Ge. In particular, I_{Chiral} and $\varphi_{0,chiral}$ can be changed when the external magnetic field is swept because they are determined by the magnetic texture of Mn_3Ge. This explains both why there is a hysteresis in the Fraunhoffer pattern and why there is a phase shift of about π in the SQUID data between Extended Data Figs. 3a and 3b.

Section S2: Quantitative comparison between the experimental data and theoretical reproduction.

In this section, we attempt to make a quantitative comparison between the theoretical prediction and the experimental data. As explicitly shown in Eq. (28), $d\frac{2JM_0}{\hbar v_F}\tau$ and $Qwd\frac{J\delta M}{\hbar v_F}\tau$ are the chirality-dependent phases arising from overall tilting of antiferromagnetic-spin texture under an OOP magnetic field. Fundamentally, the former $d\frac{2JM_0}{\hbar v_F}\tau$ gives rise to a 0-to-π phase transition in SQUID measurements whereas the latter $Qwd\frac{J\delta M}{\hbar v_F}\tau$ leads to hysteretic Fraunhofer patterns. Using the estimated value of $\left|Qwd\frac{J\delta M}{\hbar v_F}\tau\right| \approx 0.2$ from the $I_c(\mu_0 H_\perp)$ data (Fig. 1d), $d \approx 200$ nm, $w = 1.5$ µm, $\tau = \pm 1$, $Q \approx 0.1$ µm^{-1},[S11] JM_0 and $J\delta M$ are both of the order of the exchange energy $k_B T_{\text{Néel}}$ for our Mn_3Ge film ($T_{\text{Néel}} \approx 410$ K),[S12] we obtain $\hbar v_F \approx 2.9 \times 10^{-9}$ eV m. As this value is larger than estimated from the resistivity data via a *s*-band transport model[S13], it suggests a non-negligible *d*-band contribution to long-range triplet correlations in our chiral non-collinear AFM.

Most importantly, from the estimated $\left|Qw\frac{J\delta M}{\hbar v_F}\tau\right| \approx 0.001$ nm^{-1} (for $d \approx 200$ nm, Fig 1d), we can expect the 0-to-π phase transition for $d_s > 120$ nm (under application of $\mu_0 H_\perp$) and find a quantitative agreement with our experimental data (Fig. 3f, Extended Data Fig. 4).

Section S3: Unlikely self-field effect in our experimental setup.

A shift of the maxima in the Fraunhofer pattern produced by the self-field effect[S14] is given by $\frac{\Phi_S^M}{\Phi_0} = \frac{1}{4\pi}\left(\frac{d}{\lambda_J}\right)^2$, where $\lambda_J = \sqrt{\frac{\hbar}{2e\mu_0(d+2\lambda_L)J_c}}$ is the Josephson penetration depth, $\hbar$ is the reduced Planck constant, e is the electric charge, μ_0 is the permeability of free space and J_c the Josephson critical current density. Using $d = 199$ nm, $\lambda_L \approx 130$ nm and $J_c = 1.3 \times 10^5$ A/cm^{-2}, we obtain the vanishingly small $\frac{\Phi_S^M}{\Phi_0} \approx 0.005$. As this value is *approximately two*

orders of magnitude smaller than what is observed in our experimental setup $\left(\frac{\Phi_S^M}{\Phi_0} = 0.20 - 0.25\right)$, the self-field effect can be ruled out.

We further note that the almost absence of the supercurrent SV signature in the $d_s \approx 80\ nm$ JJ albeit its larger critical supercurrent being by a factor of 7 (~ 0.7 mA, Extended Data Figure 5) than the $d_s \approx 199\ nm$ JJ (~ 0.1 mA, Figs. 1d and 2d) allows one to completely exclude any feasibility of the self-field-driven hysteretic effect [S14].

References

S1. Krivoruchko, V. N. Upper critical fields of the superconducting state of a superconductor-antiferromagnetic metal superlattice. *JETP* **82**, 347–355 (1996).

S2. Kurebayashi, D. & Nomura, K. Theory for spin torque in Weyl semimetal with magnetic texture. *Sci. Rep.* **9** 5365 (2019).

S3. Bovenzi, N., Breitkreiz, M., Baireuther, P., O'Brien, T. E., Tworzydło, J., Adagideli, İ. & C. W. Beenakker, J. Chirality blockade of Andreev reflection in a magnetic Weyl semimetal, *Phys. Rev. B* **96**, 035437 (2017).

S4. Eschrig, M, Cottet, A., Belzig, W. & Linder, J. General boundary conditions for quasiclassical theory of superconductivity in the diffusive limit: application to strongly spin-polarized systems. *New J. Phys.* **17** 083037 (2015).

S5. Zyuzin, A., Alidoust, M. & Loss, D. Josephson junction through a disordered topological insulator with helical magnetization. *Phys. Rev. B* **93**, 214502 (2016).

S6. Hugdal, Henning G., Linder, J. & Jacobsen, Sol H. Quasiclassical theory for the superconducting proximity effect in Dirac materials. *Phys. Rev. B* **95**, 235403 (2017).

S7. Takeuchi, Y., Yamane, Y., Yoon, J.-Y., et al. Chiral-spin rotation of non-collinear antiferromagnet by spin-orbit torque, *Nat. Mater.* **20**, 1364 (2021).

S8. The spin rotation effect at a spin-active interface should be described with appropriate boundary conditions for the Usadel Green's functions. Such boundary conditions have already been derived for the SC/FM spin-active interfaces, see for instance M. Eschrig, A. Cottet, W. Belzig, J. Linder, *New J. Phys.* **17** 083037 (2015) and A. Cottet, D. Huertas-Hernando, W. Belzig, and Y. V. Nazarov, *Phys. Rev. B* **80**, 184511 (2009). These boundary conditions explicitly take into account the interfacial spin-precession effect and the interfacial conversion of the spin-unpolarised singlets ($S = 0$) through the spin-zero ($S = 1, m_s = 0$) to the spin-polarised triplets ($S = 1, m_s = \pm 1$). Analogous boundary conditions can be applied to the SC/AFM case, but this is beyond the scope of the present work.

S9. A. Cottet, D. Huertas-Hernando, W. Belzig & Y. V. Nazarov, Spin-dependent boundary conditions for isotropic superconducting Green's function, *Phys. Rev. B* **80**, 184511 (2009).

S10. A. Cottet et al. in preparation.

S11. T. Higo et al. Large magneto-optical Kerr effect and imaging of magnetic octupole domains in an antiferromagnetic metal, *Nat. Photo.* **12**, 73–78 (2018).

S12. Jeon, K. R. et al. Long-range supercurrents through a chiral non-collinear antiferromagnet in lateral Josephson junctions. *Nat. Mater.* **20**, 1358–1363 (2021).

S13. Wang, X. et al. Robust anomalous Hall effect and temperature driven Lifshitz transition in Weyl semimetal Mn_3Ge. *Nanoscale* **13**, 2601–2608 (2021).

S14. Barone, A. & Paterno, G. *Physics and Applications of the Josephson Effects*, John Wiley & Sons, New York (1982).
